# Exploring the temporal dynamics of speech production with EEG and group ICA

**DOI:** 10.1038/s41598-020-60301-1

**Published:** 2020-02-28

**Authors:** Niels Janssen, Maartje van der Meij, Pedro Javier López-Pérez, Horacio A. Barber

**Affiliations:** 1 Departamento de Psicología, Universidad de la Laguna, La Laguna, Spain; 2 Instituto de Tecnologías Biomedicas, Universidad de la Laguna, La Laguna, Spain; 3 Instituto de Neurociencias, Universidad de la Laguna, La Laguna, Spain; 4 Departamento de Psicología, Universidad de la Costa, Barranquilla, Colombia; 5 Basque Center on Cognition, Brain and Language (BCBL), Donostia, Spain

**Keywords:** Cognitive neuroscience, Neuronal physiology

## Abstract

Speech production is a complex skill whose neural implementation relies on a large number of different regions in the brain. How neural activity in these different regions varies as a function of time during the production of speech remains poorly understood. Previous MEG studies on this topic have concluded that activity proceeds from posterior to anterior regions of the brain in a sequential manner. Here we tested this claim using the EEG technique. Specifically, participants performed a picture naming task while their naming latencies and scalp potentials were recorded. We performed group temporal Independent Component Analysis (group tICA) to obtain temporally independent component timecourses and their corresponding topographic maps. We identified fifteen components whose estimated neural sources were located in various areas of the brain. The trial-by-trial component timecourses were predictive of the naming latency, implying their involvement in the task. Crucially, we computed the degree of concurrent activity of each component timecourse to test whether activity was sequential or parallel. Our results revealed that these fifteen distinct neural sources exhibit largely concurrent activity during speech production. These results suggest that speech production relies on neural activity that takes place in parallel networks of distributed neural sources.

## Introduction

It is now well understood that the production of speech relies on neural activity in a wide range of different areas of the brain e.g.^1–7^. How this activity is coordinated over time such that it results in fast and fluent speech remains largely unknown. Therapy design for various pathologies such as aphasia, dysarthria and stuttering requires first a good understanding of the workings of speech production under non-pathological circumstances. Here we examined the activation dynamics of different areas of the brain while speech was produced. Specifically, speech production was elicited using a picture naming task in which participants overtly produced single words in response to visually presented objects. Previous Positron Emission Tomography (PET) and functional Magnetic Resonance Imaging (fMRI) studies have repeatedly shown that picture naming yields activation in occipital, temporal, frontal and parietal areas of the cortex, as well as in the striatum, thalamus and brain stem of the subcortex e.g.^8–12^. Although these studies are informative about the location of brain activity underlying picture naming, other techniques such as Magnetoencephalography (MEG) and Electroencephalography (EEG) are needed to examine the precise temporal dynamics of neural activity underlying the task. With respect to this issue, previous MEG studies have concluded that neural activation underlying picture naming proceeds from posterior to anterior areas of the brain in a sequential manner e.g.^13–18^. Here we attempted to validate this conclusion from MEG studies by using a different analysis approach that relied on EEG data.

Before we start we should highlight that our study addressed a specific hypothesis about the activation dynamics of different brain regions during speech production. This topic bears resemblance to but is different from topics addressed by recent other studies in the field of language production and EEG. These studies have focused on questions about the timecourse of cognitive/linguistic properties such as semantics, lexical access and phonology e.g.^19–25^, and not on the activation dynamics of brain regions. We will come back to this issue in the General Discussion.

Previous MEG studies on the activation dynamics of speech production in the picture naming task have all relied on a similar analysis approach. For example, in a seminal study by Salmelin *et al*.^16^, participants were required to overtly produce the names of around 100 presented objects that appeared for 100 ms every 5 s on a computer screen. The analysis approach involved the following three steps. First, a grand average stimulus-locked epoch was calculated for each participant. This epoch contained the activation profile across all trials for a time period of 1.5 s following stimulus onset for each sensor. Next, for each participant, the time-varying topographic maps corresponding to the stimulus-locked epochs were scanned for dipolar map profiles. The cortical location of each discovered dipole was then estimated using Equivalent Current Dipole (ECD) fitting procedures^26^. This led to the identification of approximately 10 neural sources for each participant. Finally, the point in time when a given cortical source was activated was based on the peak latency of fitted dipole time courses. This peak latency was then classified into one of three time bins. Salmelin *et al*. observed that there were sources in occipital areas in a timebin that spanned from 0 to 200 ms, in temporo-parietal areas between 200 to 400 ms, and in frontal areas between 400 to 800 ms. Other MEG studies using this procedure have found comparable results e.g.^14,17^. Given that picture naming typically takes around 700 ms, these results have led to the conclusion that during picture naming, activation progresses sequentially from occipital input areas to higher level temporo-parietal areas, and finally to frontal motor output areas.

However, there are four important problems with this analysis approach that undermine this conclusion. First, a major aspect of the analysis approach relied on the identification of neural sources from the detection of dipolar maps in the epoched MEG data. However, topographic maps of original epoched data usually contain a mix of dipoles which may produce uncertainties in the source localization procedure^27^. In addition, the analysis approach did not verify that the obtained neural sources affected performance in the task and therefore their inclusion in the analyses may have been spurious. Third, the procedure relied on the peak latency as the primary identifier for the point in time when a particular neural source was active during speech production. However, it is not obvious that peak latency is a representative marker for characterizing the dynamics of a source’s neural activity. Consider, for example, the visual evoked potential which is a complex of peaks and troughs (N1, P1, N2) that appears in early visual processing e.g.^28,29^. It would be misleading to characterize activity of this potential by a single peak latency. Finally, to obtain grand average timecourses, amplitudes across all trials were averaged. However, it is well known that there is a substantial amount of trial-by-trial response latency jitter in the picture naming task^30,31^. Simply averaging across all trials may have led to imprecise characterization of the activation dynamics underlying the picture naming task. In short, these problems call into question the claim that activity in the picture naming task proceeds from posterior to anterior areas of the brain in a sequential manner.

In the experiment reported below participants produced the names of 100 visually presented objects while their EEG was recorded. In order to examine the temporal dynamics of brain regions involved in the picture naming task we relied on the following analysis approach. Instead of finding dipolar maps in the original observed data, we attempted to identify dipolar maps generated by a procedure called Independent Component Analysis (ICA). One advantage of ICA is that the obtained topographic component maps often contain single dipoles^27^. The neural sources of these dipolar maps can be estimated in a relatively straightforward manner using ECD fitting procedures^26^. In addition, in contrast to the aforementioned MEG studies, we ensured that the component activity of these discovered neural sources was affecting performance in the picture naming task. We did this by regressing the picture naming latency onto the independent component’s amplitude in the task at each respective time point on a by-trial basis^32^. Furthermore, we did not rely on peak latency measures. Instead, we calculated the onset and offset time of neural activity of each neural source based on the time at the full-width half maximum (FWHM) values for each component and then computed their percentage of temporal overlap. Finally, we examined the degree to which our estimates were affected by the latency jitter that is inherent in picture naming. To this end we performed an analysis with all trials as well as an analysis that only included those trials that fell within 0.25 standard deviations of the mean latency of the experiment.

We expected that if the brain regions involved in picture naming are active in sequence, we would find little overlap in the activity between any of the neural sources obtained from the ICA.

## Methods

### Participants

Thirty native speakers of Spanish took part in the experiment. Participants were students at the Universidad de La Laguna, and received course credit or were paid 10 Euro. The experiment was approved by the Ethics Committee of the University of La Laguna (CEIBA) and was executed in accordance with their guidelines. Informed consent was obtained from all participants in the study. The data from five participants were discarded due to excessive noise in the recordings (i.e., >30% rejection of trials). The analyses reported below therefore contained 25 participants.

### Task details

One hundred images were selected from a standardized picture database commonly used in speech production research^33^. The images depicted black and white line-drawings of common, every-day objects that had high name agreement (>90%). In addition, the images varied in terms of image complexity (mean = 16147; sd = 8756), and had names with variability in log word frequency (mean = 3.23; sd = 2), and word length in phonemes (mean = 6; sd = 2). Pictures were standardized with respect to their size such that each picture fit within a rectangle of 300 × 300 pixels. Each picture appeared in the center of the screen as a white line-drawing on a black background. Pictures were presented on a CRT monitor using a refresh rate of 80 Hz in a 1024 × 768 resolution. Response latencies and vocal responses were recorded using an electret large diaphragm microphone (IMG stage line, ECM-140) placed at an average distance of 100 cm away from the participant. Stimulus presentation, voice-key detection, and digitization of vocal responses were controlled by a computer running Presentations (Neurobs, V14.5).

In the experiment, participants were instructed to produce the name of presented pictures. On each trial, a fixation cross was presented for 700 ms, followed by the target picture for 1500 ms. The next trial started after a blank screen presentation for 2000 ms. All 100 pictures were named three times; once before familiarization, once during familiarization, and once after familiarization. During familiarization, the pictures were presented with their names and participants were instructed to name each picture with its corresponding name. EEG was not recorded during this familiarization stage. This study does not consider the role of familiarization and therefore data from before and after familiarization were analyzed jointly, leading to 200 trials per participant. Note, however, that familiarization reduces naming latencies which introduces a naming latency jittering in the data. Consequently, we considered the impact of familiarization in complementary analyses reported below.

### EEG Recordings

The software used to register the EEG signal was BrainVision Recorder (Brain Products, V1.03). The continuous EEG signal was recorded with 32 Ag/AgCl electrodes embedded in an elastic cap using Easycap (http://www.easycap.de) that followed the standard 10–20 system. On-line recordings were referenced to the left mastoid. The signal was amplified (BrainAmp amplifiers) and digitized at a sampling rate of 250 Hz, with a 0.01–100 Hz band pass filter. The horizontal electrooculogram (EOG) was measured by placing two electrodes at the outer canthi, and the vertical EOG was measured with two electrodes placed above and below the left eye. Electrode impedance was kept below 5 k*Ω* for all electrodes.

### EEG Pre-processing

Pre-processing was performed in EEGLAB (v2019_1;^34^). After data acquisition, the data were offline re-referenced using the right mastoid. The electrode corresponding to the right mastoid was subsequently removed from the data, leaving 31 electrodes. Next, low frequency oscillations in the raw EEG data were removed using the eeglab default highpass filter at 1 Hz. This particular highpass filter value was chosen as an optimal compromise between maximizing the SNR and detection of ICs with ICA^35^, and minimizing the impact of data alterations^36–39^. High frequency oscillations were subsequently removed by a lowpass filter at 50 Hz. Next, we epoched the continuous EEG data for each participant into 200 extended epochs with a duration of 2000 ms each (500 ms before stimulus onset until 1500 ms after stimulus onset), with each epoch corresponding to a picture naming trial. The following step in pre-processing consisted of automatic artifact rejection. Specifically, an epoch was removed if it contained an amplitude value that was outside a given negative or positive threshold. Determination of these thresholds was done by automatically lowering or raising the threshold value such that it resulted in the removal of approximately 10% of the epochs for a given participant. In order to prevent this procedure from resulting in very high thresholds (when data was noisy), a maximum value for each threshold was instigated (±200 *μV*). For some participants this resulted in a rejection rate of more than 10%. In addition, we removed those epochs for which the participant did not produce the correct target stimulus, or for which the target naming latency was outside the [300, 1500] ms range. In total, the average number of rejected epochs was 16% (range 11–27%).

**Figure 1:**
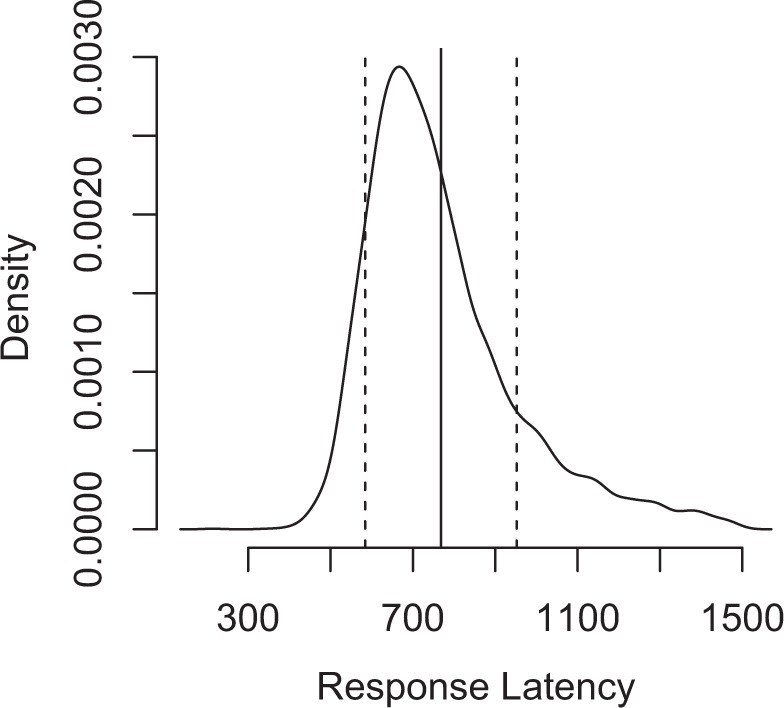
Distribution of response latencies in the experiment as a smoothed density function. On x-axis are plotted the naming latencies (in ms), and on the y-axis are plotted the relative frequencies. Also plotted are the mean naming (vertical solid line) and latencies at 1 standard deviation (dotted lines).

The final step in preprocessing was the reduction of the epoch length to 600 ms. Specifically, each epoch was reduced to a time window from −100 to 500 ms. This epoch length was chosen in order to avoid the influence of artefacts due to articulation that is inherent in an overt speech production task. Previous speech production EEG studies have shown that EEG data are strongly affected by the onset of speech due to the movement of muscles in face which may contaminate the EEG signal^40^. Given that only 1.6% of latencies were <500 ms (see Fig. 1), we assumed that this epoch length was sufficiently long to lead to good ICA estimations (see below) while also minimizing the contamination produced by speech motor artefacts.

#### Group temporal ICA

ICA is a blind-source separation technique that attempts to discover underlying sources from the linear mixing of these sources in the observed data^41^. In contrast to fMRI studies where spatial ICA is more common^42^, electrophysiological studies have generally relied on a version of ICA called temporal ICA^43^. In temporal ICA (tICA), it is assumed that the data matrix *X* with *m* electrode rows and *n* timepoint columns is the product of a mixing matrix *A* and a sources matrix *S* (*X* = *AS*). Matrices *A* and *S* can be calculated by an iterative procedure in which the non-gaussianity of the sources found in the rows of matrix *S* is maximized by estimation of a weight matrix *W* such that *S* = *WX*^44^. After *S* has been found, matrix *A* can be found by the calculation of *W*^−1^. Group tICA involves an identical procedure, except that the data matrix *X* now contains all individual participant data matrices concatenated by columns, leading a very wide data matrix [see, for example^45^, for a more complete formal description of group ICA in EEG].

In our data, matrix *X* corresponded to a concatenation of all correct trials for all participants. The size of this matrix was 31 × *k*, where 31 refers to the number of electrodes, and *k* was equal to the total number of correct epochs across all participants times the number of time points per epoch = 4217 × 150 = 632, 550. This whole matrix was then entered into the ICA. Following others^46^, we used all 31 electrodes in the analyses including those related to eye movements. Although it is common to use an initial principal component analysis to reduce to size of data matrix *X*, we decided to skip this step based on previous research that has shown improved component estimation under such circumstances^47^. We used the INFOMAX version of ICA in the pre-compiled binary FastICA implementation that is available in the Linux version of EEGLAB^48^. We will refer to the component sources as revealed by ICA as Independent Components (ICs). Note that this use of the word ’component’ differs from other uses of this word in the ERP literature where it typically refers to a large deflection in the ERP amplitude (e.g., N400 component).

#### Dipole fitting

Each of the 31 detected ICs had a corresponding topographical scalp map that often contained a single dipole^27^. For those components that contained a single dipole, its source activity could be estimated in a relatively straightforward manner using ECD modeling^27^. ECD modeling of each IC was performed using the DIPFIT plug-in (v3.3) available in EEGLAB. ECD modeling is a procedure that fits a dipole model with parameters for location, orientation and amplitude to the data using a least squares algorithm. For forward modeling we chose the standard spherical head model. This model is based on a four-shell model of electrical conductance across four layers of the brain (skin, skull, CSF, and cortex). For each dipole we obtained its location, orientation, and amplitude in MNI space (as a 3D vector), as well as its Residual Variance (a goodness of fit estimate). This version of Dipfit also provided anatomical inferences of each fitted dipole using the Harvard-Oxford cortical and sub-cortical probabilistic Atlas^49^.

#### Classification of ICs

Next, we classified each IC as signal (i.e., having a neural source) or noise (i.e., having a non-neural source). This classification was made on the basis each IC’s average component timecourse, topographic map and fitted dipole map. Specifically, ICs were classified as signal when they met all of the following three citeria: 1) if their timecourse revealed clear synchronized activity that was present on all trials, 2) if the topographic map was clearly dipolar, and 3) if the residual variance of the fitted dipole was ≤6%. Importantly, note that this procedure also addresses the issue of artefact removal. Specifically, to the degree that ICA successfully separates noise components (e.g., eye blinks, electrical signal noise, muscle artifacts) from signal components, the signal components will be statistically unrelated to the noise components e.g.^50,51^. In other words, given that our further downstream analyses were performed only on ICs identified as signal they will be effectively free of artefacts.

#### Relationship between IC and task performance

In the next step of the analysis we determined whether a given IC identified as signal was involved in the picture naming task. We reasoned that if an IC was involved in the task, its component activity should be predictive of the naming latency in the task. Note that establishing whether neural activity plays a causal role in a task is a complex issue that is relevant to many branches of neuro-imaging^52^. To assess this, we fitted a linear regression model using the picture naming latency as the dependent variable and the component amplitude as the predictor for each time point in the 600 ms epoch. This kind of analysis is commonly known as a running t-test^32,53^. Note that for these analyses we used all available trials that were not averaged by-participants, meaning that our degrees of freedom was large (>4000). In addition, for a given component we computed 150 t-tests (i.e., at each time point across the −100 to 500 ms range). To protect against false-positives, we used the procedure outlined by^54^ to compute an appropriate null level for hypothesis testing. Using an in-house implemented version of their procedure with 10000 simulations, N = 25, timepoints = 150, autocorrelation = 0.9, and alpha = 0.0005, we determined that significance at this alpha level can be assumed if there are at least 5 consecutive timepoints with p values below the alpha level. Our assumption of at least 10 consecutive timepoints with p-values below the alpha level of *p* < 0.0005 can therefore be considered a strongly conservative estimate of significance.

#### Temporal overlap between ICs

The next step of the analyses involved determining the percentage of overlap in the activity time courses of the various signal ICs. Specifically, for each IC we first computed the group-level average component time course by averaging across all epochs from all participants. We then computed for each IC, the onset timepoint of the activity in a component as the first moment in time (relative to stimulus onset) when a component’s amplitude first reached 50% of the component’s absolute maximum amplitude value (i.e., the component’s time to FWHM). The offset of component activity was computed in an identical manner by starting at the end of the time course and working backwards. The percentage of activity overlap (*AO*) between one target IC*A* relative to another IC*B* was then computed as follows

1 $$AO=\frac{I(a,b)}{{T}_{a}},$$

where *a* and *b* referred to the sequence of timepoints from the obtained activity onset to the activity offset for ICs *A* and *B* respectively, *I*(⋅) computes the intersection of timepoints between vectors *a* and *b*, and *T*_*a*_ refers to the total number of timepoints (from onset to offset) for target IC *A*. This formula therefore computed the sequence of overlapping timepoints between one target IC and another IC relative to the total number of timepoints in the target IC. Note that this will result in asymmetrical overlaps because the overlap of a given target *a* with respect to *b* may be different than the overlap of *b* with respect to *a*. Finally, we computed the overlap in milliseconds (ms) as the difference between the offset and onset of the intersection timepoints.

#### Latency jitter

In the final step of the analyses we estimated the effect of latency jitter on the component time courses^31^. To do this, we re-computed the above analyses using only those trials on which the naming latency fell within ±0.25 standard deviations from the mean naming latency in the experiment. Latency jitter may blur the precise onset and offset of the component time courses and therefore yield incorrect results. Note that this aspect of the analyses takes into account the potential jittering in naming latencies due to the familiarization procedure. By restricting the analyses to a set of naming latencies that are similar one minimizes the effects of latency jitter on the component time courses.

## Results

### RT results

All digitized vocal responses were manually checked using CheckVocal^55^. We removed those response latencies that corresponded to an incorrect response, as well as those which were implausible in a picture naming experiment (those smaller than 300 or larger than 1500 ms). From a total number 5000 response latencies (i.e., 25 subjects * 200 trials), 256 latencies were removed (5.1%). As explained above, trials were also removed based on EEG properties. The final number of trials that was included in the analysis was 4217 (84.3%). In this final set, the mean picture naming latency was 768 ms, and the standard deviation was 184 ms. For a graphical presentation of the distribution of response latencies in the experiment using a smoothed histogram plot, see Fig. 1.

### EEG results

**Figure 2:**
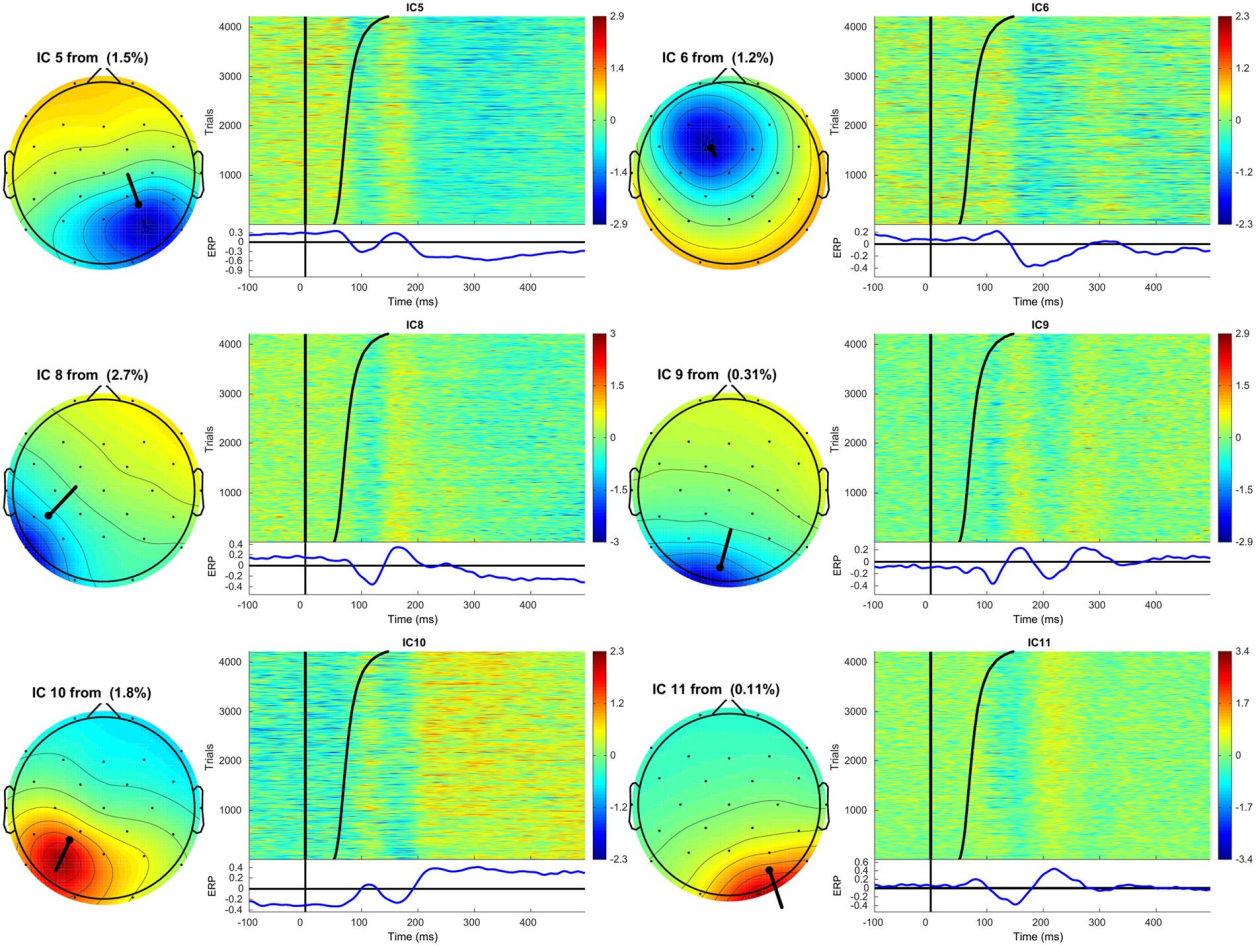
Topographic map and corresponding ERPimage of ICs 5,6,8,9,10, and 11. The topographic maps displays each dipole’s location and moment (Residual Variance in the map’s title). The ERPimage contains all the component time courses for each trial in the experiment. The trials are sorted on a linear transformation of their naming latency (denoted by the curved black line), with shorter naming latencies at the bottom of the graph. Below is presented the average component time course across all trials. Note how this component time course is present across the majority of trials in the experiment, contains a single dipole and has RV < 6%.

**Figure 3:**
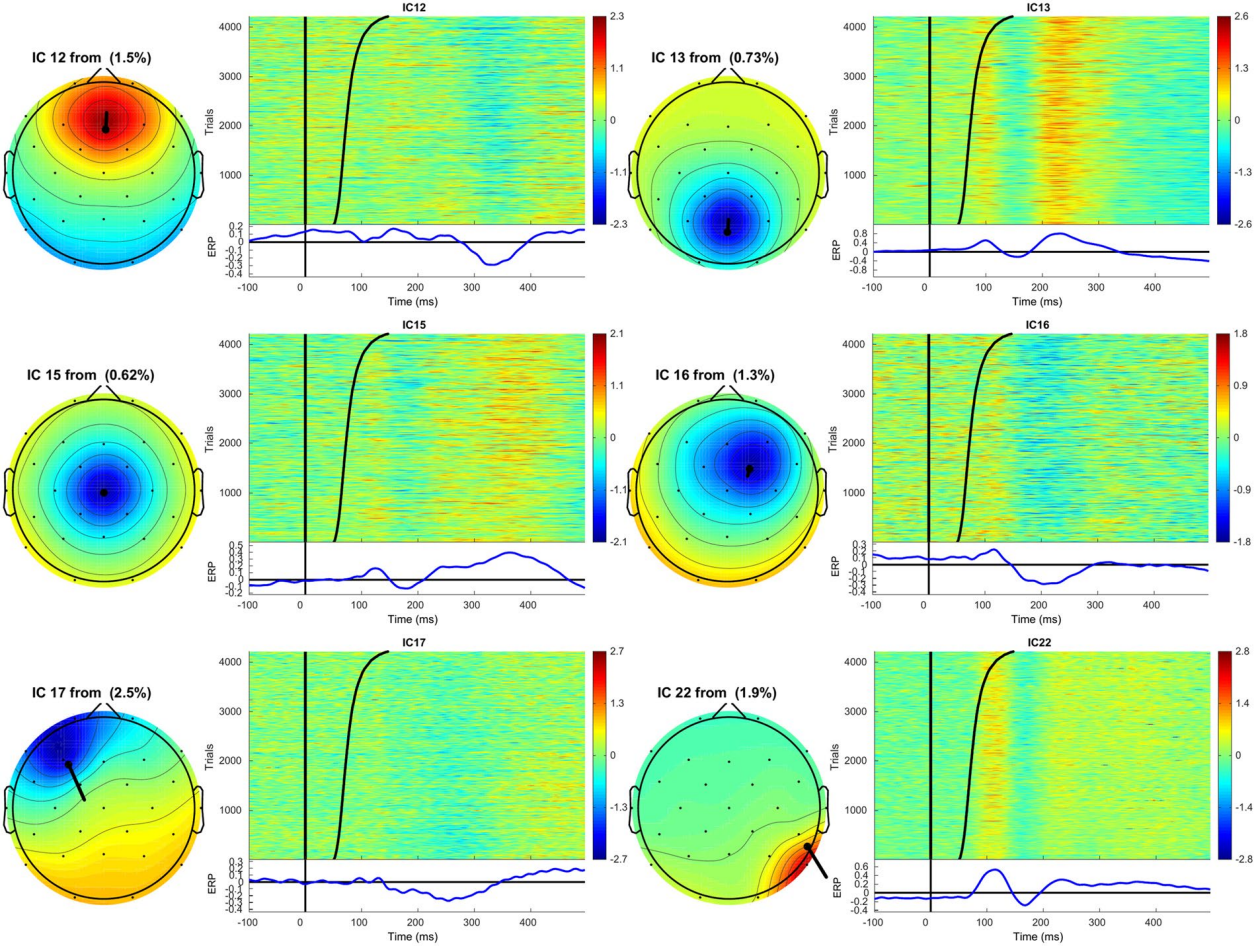
Topographic map and corresponding ERPimage of ICs 12, 13,15,16,17, and 22. See caption Fig. 2 for details.

**Figure 4:**
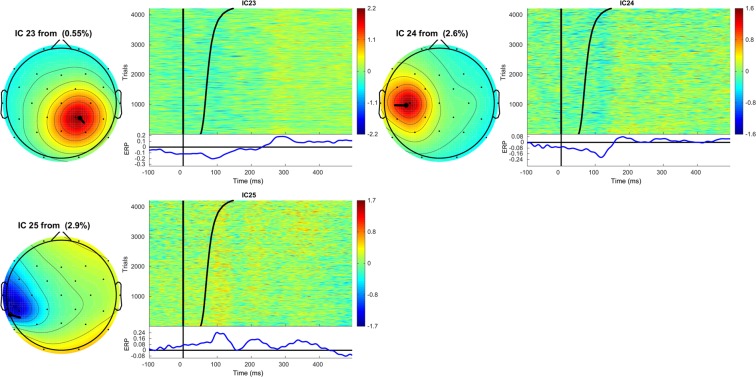
Topographic map and corresponding ERPimage of ICs 23, 24 and 25. See caption Fig. 2 for details.

After the group temporal ICA, manual inspection of the 31 ICs revealed a set of 15 ICs that met our previously defined criteria for an IC that is likely a neural source of activity (see above), and was involved in the picture naming task. These ICs were IC5, IC6, IC8, IC9, IC10, IC11, IC12, IC13, IC15, IC16, IC17, IC22, IC23, IC24 and IC25. Figures 2, 3, 4 display the topographic maps and dipoles of each IC. See also Supplementary Figs. S1–S3 for an overview of all ICs that were considered noise using the three criteria outline above. Figures 2, 3, 4 also displays each IC’s corresponding timecourse for each trial (sorted by naming latency) across the full range of timepoints that formed the basis of the ICA, and the timecourse averaged across all trials extracted from the erpimage function of EEGLAB^56^. Note in Fig. 2 how component amplitudes display deflections that are present across most trials in the experiment, the topographic map of each detected IC has a single dipole, and how the residual variance of each fitted dipole is ≤6%).

**Figure 5:**
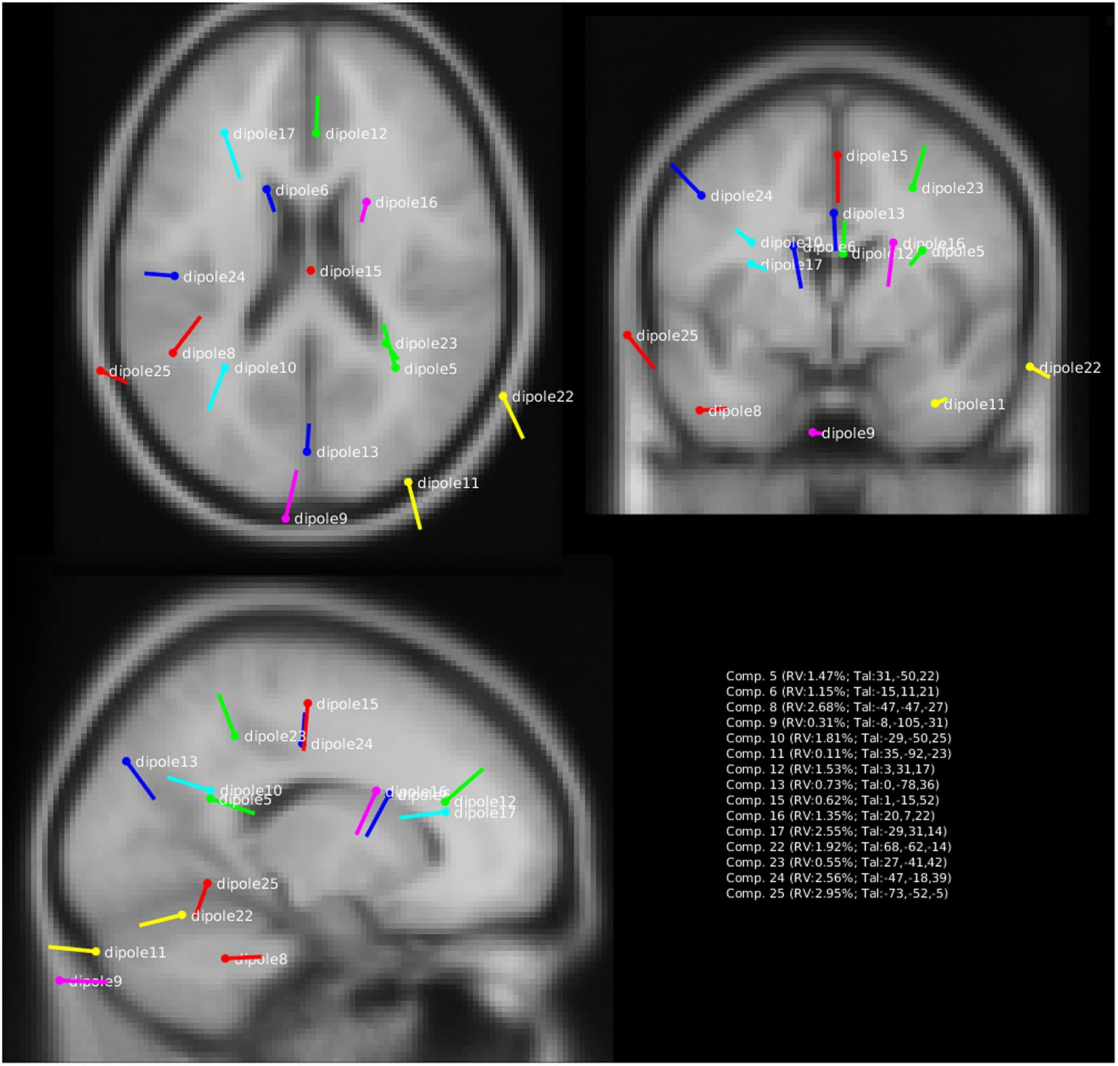
Source localizations of the fifteen components in the experiment using equivalent current dipole source fitting. Plotted are the fifteen components with clear dipolar topographic maps (see Figs. 2–4). For each component we also list the Residual Variance (RV) between the model prediction and the observed data, as well as the anatomical coordinates in Talairach space (between brackets). Note how sources are localized in many different areas of the brain, including occipital, parietal, temporal and frontal areas.

**Figure 6:**
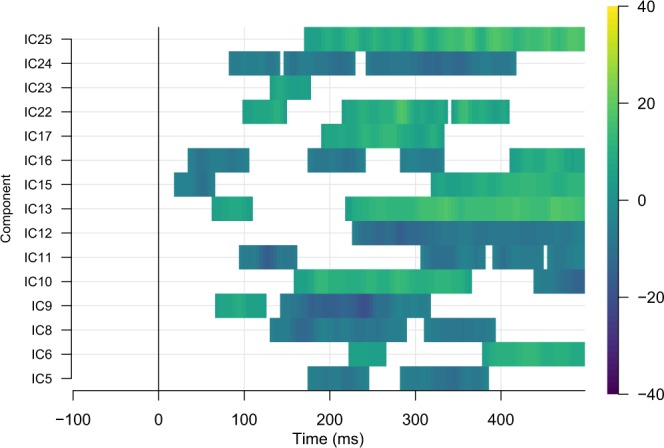
Relationship between component amplitude and naming latency across the component time course. Plotted here are the t-values that express the degree to which naming latencies were predicted by the amplitude at each time point and for each individual component. Warm (yellow) colors denote positive t-values, whereas cool (magenta) colors denote negative t-values. Thus, for example, the green values at around 100 ms for component C9 indicate that at around 100 ms after picture presentation, amplitudes of component C9 are predictive of the specific naming latency. Regression analyses took place on trial-based data. To protect against false-positives due to the high number of statistical tests, only t-values > 4 for ten consecutive time points are plotted.

 Figure 5 displays the estimated location of the cortical sources that gave rise to the topographic maps of each IC as obtained by ECD modeling^26^. Table 1 lists each IC with the anatomical interpretation of its nearest identifiable gray matter source using the Harvard-Oxford probabilistic atlas^49^ as was provided by the Dipfit plugin. As can be seen in Table 1, sources in a wide variety of cortical locations were found. Specifically, sources were found in occipital (IC13, IC9, IC11), parietal (IC5,IC24,IC10,IC23), temporal (IC22, IC25, IC8) and frontal (IC6,IC16,IC17,IC15,IC12) regions. In addition, Fig. 6 revealed the correlation of each of these components’ amplitude with the naming latency across each timepoint in the epoch. As is clear from this figure, the component amplitude was predictive of the trial’s naming latency across a wide range of timepoints suggesting the involvement of these components in the task (see Fig. S4 for an overview of the relationship between naming latency and all ICs).

**Table 1:** Estimated source localization of each Independent Component using the group-level data. Coordinates in MNI space (XYZ) as well as detected onset, offset and their difference (in ms) of activity in the component timecourses (see text for further details).

IC	Estimated Localization	X	Y	Z	onset	offset	diff
IC5	inferiorparietal R	−30	−33	17	88	496	408
IC6	caudalanteriorcingulate L	23	16	19	152	412	260
IC8	inferiortemporal L	−24	51	−32	92	496	404
IC9	lateraloccipital L	−72	8	−39	104	488	384
IC10	inferiorparietal L	−30	31	20	108	496	388
IC11	fusiform R	−62	− 38	−31	120	240	120
IC12	caudalanteriorcingulate R	40	− 2	17	296	376	80
IC13	cuneus L	−55	0	29	92	496	404
IC15	paracentral R	−2	0	47	240	436	196
IC16	caudalmiddlefrontal R	19	−21	20	156	496	340
IC17	rostralmiddlefrontal L	40	33	13	168	496	328
IC22	middletemporal R	−36	−73	−19	88	404	316
IC23	superiorparietal R	−23	−29	37	252	496	244
IC24	supramarginal L	−4	51	35	88	496	408
IC25	middletemporal L	−30	78	−9	88	480	392

^1^IC = Independent Component.

**Figure 7:**
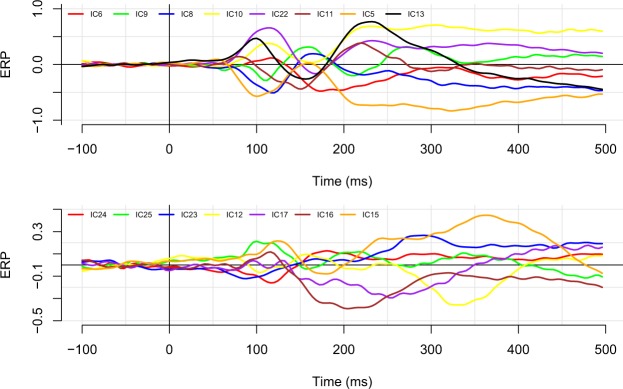
Average timecourses for components 6, 9, 8, 10, 22, 11, 5, and 13 (top panel), and for components 24, 25, 23, 12, 17, 16, and 15 (bottom panel). Components are ordered on the basis of the their degree of explained variance to enable different scales between the two graphs. Note the large degree of overlap between the various component time courses.

In Figure 7 we plotted the average component timecourse for each component in a single image. Visual inspection of these images suggested that the neural activity underlying each IC largely overlaps in time. Quantitative analyses revealed that the average onset (i.e., the timepoint at FWHM) of component activity was 142 ms after stimulus presentation, and the average offset was 453 ms. As can be seen in Table 1, most ICs were active for a substantial portion of the epoch with activities ranging between 80 ms for IC12 and 408 ms for ICs 24 and 5. Table 2 shows the percentage overlap of the component timecourses for each pair of ICs. The average overlap was 80.1%, with the maximum overlap of 100% between various components, and the minimum overlap of 0% between components IC12 and IC11.

**Table 2:** Ratios (top panel) and milliseconds (bottom panel) of group-level activity overlap between the time courses of each Independent Component.

	5	6	8	9	10	11	12	13	15	16	17	22	23	24	25
5	1.00	1.00	1.00	1.00	1.00	1.00	1.00	1.00	1.00	1.00	1.00	1.00	1.00	1.00	1.00
6	0.64	1.00	0.65	0.68	0.67	0.74	1.00	0.65	0.88	0.76	0.75	0.80	0.66	0.64	0.67
8	0.99	1.00	1.00	1.00	1.00	1.00	1.00	1.00	1.00	1.00	1.00	0.99	1.00	0.99	0.99
9	0.94	1.00	0.95	1.00	0.98	1.00	1.00	0.95	1.00	0.98	0.98	0.95	0.97	0.94	0.96
10	0.95	1.00	0.96	0.99	1.00	1.00	1.00	0.96	1.00	1.00	1.00	0.94	1.00	0.95	0.95
11	0.30	0.35	0.30	0.32	0.32	1.00	0.00	0.30	0.02	0.26	0.23	0.39	0.00	0.30	0.31
12	0.20	0.32	0.21	0.22	0.21	0.00	1.00	0.21	0.42	0.24	0.25	0.26	0.34	0.20	0.21
13	0.99	1.00	1.00	1.00	1.00	1.00	1.00	1.00	1.00	1.00	1.00	0.99	1.00	0.99	0.99
15	0.49	0.67	0.49	0.52	0.51	0.03	1.00	0.49	1.00	0.58	0.60	0.53	0.76	0.49	0.51
16	0.83	0.98	0.84	0.87	0.88	0.71	1.00	0.84	1.00	1.00	1.00	0.79	1.00	0.83	0.83
17	0.81	0.94	0.81	0.84	0.85	0.61	1.00	0.81	1.00	0.97	1.00	0.75	1.00	0.81	0.80
22	0.78	0.97	0.77	0.78	0.77	1.00	1.00	0.77	0.84	0.73	0.72	1.00	0.63	0.78	0.81
23	0.60	0.62	0.61	0.62	0.63	0.00	1.00	0.61	0.94	0.72	0.75	0.49	1.00	0.60	0.59
24	1.00	1.00	1.00	1.00	1.00	1.00	1.00	1.00	1.00	1.00	1.00	1.00	1.00	1.00	1.00
25	0.96	1.00	0.96	0.98	0.96	1.00	1.00	0.96	1.00	0.95	0.95	1.00	0.94	0.96	1.00
5	408.00														
6	260.00	260.00													
8	404.00	260.00	404.00												
9	384.00	260.00	384.00	384.00											
10	388.00	260.00	388.00	380.00	388.00										
11	120.00	88.00	120.00	120.00	120.00	120.00									
12	80.00	80.00	80.00	80.00	80.00	0.00	80.00								
13	404.00	260.00	404.00	384.00	388.00	120.00	80.00	404.00							
15	196.00	172.00	196.00	196.00	196.00	0.00	80.00	196.00	196.00						
16	340.00	256.00	340.00	332.00	340.00	84.00	80.00	340.00	196.00	340.00					
17	328.00	244.00	328.00	320.00	328.00	72.00	80.00	328.00	196.00	328.00	328.00				
22	316.00	252.00	312.00	300.00	296.00	120.00	80.00	312.00	164.00	248.00	236.00	316.00			
23	244.00	160.00	244.00	236.00	244.00	0.00	80.00	244.00	184.00	244.00	244.00	152.00	244.00		
24	408.00	260.00	404.00	384.00	388.00	120.00	80.00	404.00	196.00	340.00	328.00	316.00	244.00	408.00	
25	392.00	260.00	388.00	376.00	372.00	120.00	80.00	388.00	196.00	324.00	312.00	316.00	228.00	392.00	392.00

**Figure 8:**
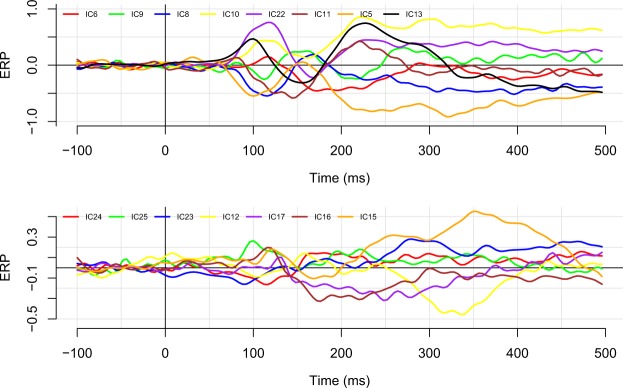
The effect of latency jitter on the component timecourses. As in Fig. 7, we plot again the average timecourses for components 6, 9, 8, 10, 22, 11, 5, and 13 (top panel), and for components 24, 25, 23, 12, 17, 16, and 15 (bottom panel). In this plot, averages are based on a subset of the data that includes only those epochs on which the naming latency was within 0.25 standard deviations from the overall mean. Although it is clear that there is more noise in the data (there are fewer data points), the overall overlapping structure is preserved.

To examine the impact of latency jitter on these data, we performed the same analyses on a subset of data that was restricted to epochs on which the latency was highly similar. Specifically, we selected those trials whose naming latency fell within ±0.25 standard deviations from the mean. This resulted in 797 trials that had naming latencies within a 80 ms range (i.e., trials had naming latencies faster than 730 and slower than 810 ms). For this specific set, the average percentage overlap between IC timecourses decreased slightly to 79.9%. A graphical presentation of the component timecourses using the restricted set of trials is presented in Fig. 8.

### Complementary analyses

**Figure 9:**
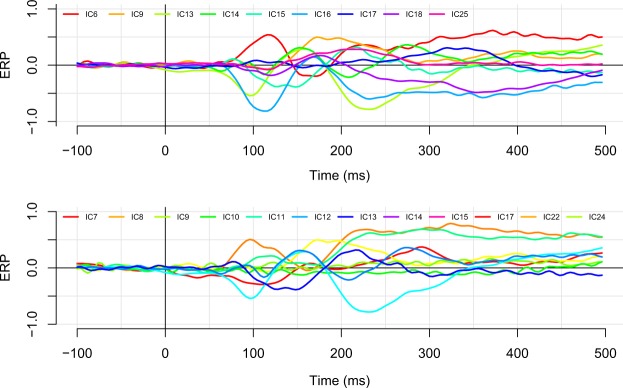
Average component timecourses of ICs that met our previously established criteria before (top panel) and after (bottom panel) familiarization (see also Supplementary Figs. S5–S12 and Supplementary Tables S1 and S2). Note that similar but not identical sets of ICs met our criteria before and after familiarization, and that there was in both cases a large degree of temporal overlap.

In further analyses we considered the impact of our familiarization procedure on the percentage of overlap between IC timecourses. Behavioral analyses revealed that naming latencies were significantly slower before than after familiarization (808 ms vs 726 ms, p <  0.0001). One potential concern is that familiarization may alter neural processing in a qualitative way thereby undermining our justification of performing a joint analysis that collapses across familiarization. We ran separate Independent Component Analyses on the preprocessed trials prior to familiarization and on those after familiarization. We then classified components from the two Independent Component Analyses as signal or noise following the three criteria listed above. Supplementary Figs. S5–S8 represents an overview of all ICs detected prior to familiarization, and Supplementary Figs. S9–S12 represents an overview of the ICS detected after familiarization. Tables S1 and S2 present the percentage and millisecond overlap between ICs before and after familiarization. As can be seen in Fig. 9, similar but not identical sets of ICS met our criteria for the trials before and after familiarization presumably reflecting the fact that different sets of the trials are used before and after familiarization. The average activity overlap of these ICs was 70% before familiarization and increased to 80% after familiarization. We therefore concluded that familiarization leads to quantitative but not qualitative changes in neural processing, and that there is a substantial amount of overlap in the activity profiles of sources detected prior to familiarization as well as after familiarization.

In addition, we attempted to assess whether the method of overlap calculation led to inflated estimates of concurrent activity. Specifically, in this analysis, we only considered those sources that had a transient activation profile (ie., an activation duration of ≤ 350 ms during the epoch, see Table 1). One could argue that such transient sources are active only for a short period of time during the task and may therefore reveal the sequential activation pattern predicted by the MEG studies. Eight ICs met our selection criteria (ICs 6, 11, 12, 15, 16, 17, 22, and 23). In addition, to avoid inflated estimates of overlap we took two further precautions: First, to avoid inflated overlap due to nearby sources, we only computed overlap between sources from the four major anatomical lobes (occipital, parietal, temporal, and frontal). Second, we only considered a single source for each of these four major lobes. These conditions led to four possible permutations of four sets of sources: occipital (IC11), parietal (IC23), temporal (IC22), and frontal (either IC6, 12, 16, or 17). In other words, we computed percentage overlap between these four different sets of sources, where there was only a single transiently activated source in each of the four major anatomical lobes. Under the predictions of the MEG study we expected to see a sequential pattern of activity with a minimal amount of overlap. The obtained overlaps for these four sets was (48%, 45%, 43%, 43%) with an average of 45%. We discuss this issue further below.

## General Discussion

The current study examined the temporal dynamics of brain activity during speech production. Participants were asked to overtly produce the names of depicted objects while their naming latencies and EEG were recorded. We used group temporal ICA to obtain temporally independent component timecourses and their corresponding topographic maps. Source estimations of dipolar maps revealed 15 brain regions that were classified as signal using three explicit criteria. We verified that the component activity of these sources affected performance in the task using regression analyses. We found that activity in these fifteen brain regions was largely concurrent: Overlap in activity defined in terms of an amplitude’s FWHM between the fifteen regions varied from 0% to 100% (average 80.1%). This overlap was found even when latency jitter across all trials was taken into account.

These results are at odds with the prediction from previous MEG studies that brain activity during speech production in the picture naming task proceeds from posterior to anterior areas of the brain in a sequential manner e.g.^13–18^. One explanation for this difference relates to the different analysis methods used to decide whether and when a particular region in the brain was active during the picture naming task. Specifically, previous MEG studies have relied on source localization from raw epoched MEG data which may have led to imprecise source localization. Moreover, previous MEG studies relied on the classification of peak latencies into three discrete timebins to determine when a particular region is active during the task. However, classification of peak latencies into discrete timebins may produce inaccurate estimates of when a particular brain region is active during a task. To illustrate this issue, consider, for example, ICs 22 and 16 (see Fig. 3). Independent Component 22 was localized in the temporal region and IC16 was located in the lateral Frontal region (see Fig. 5). A peak latency approach may lead to the classification of IC22 in an early timebin (0–200 ms) which may be earlier than the peak latency found for IC16. However, it does not seem accurate to claim that IC22 was active only in a timebin between 0 and 200 ms when its activation profile clearly extends beyond the 200 ms boundary (see Fig. 3). Note the long onset for this component is not simply a product of latency smoothing: It is also found in the analyses with the restricted latencies (cf., Figs. 7 and 8). By contrast, in the current approach, component IC22’s onset activity was detected at 88 ms and its offset at 404 ms (see Table 1) and therefore overlapped significantly with other components (e.g., with IC16 79% or 248 ms). Thus, differences in the methodology of determining whether and when a given brain region is active may explain why our results differ from those found in previous studies.

One potential concern with this line of reasoning is that we have overestimated the degree of temporal overlap during picture naming because we have included in our analyses source regions that show activity across the entire epoch. For example, as can be seen Table 1, there are several ICs that show activity across the entire epoch (e.g., IC5, IC9, IC24, IC25). One potential problem is that such regions may reflect general cognitive processes such as visual attention that are not directly related to the picture naming task. In such a scenario, the computation of overlap does not reflect only processes involved in picture naming and may therefore lead to inflated estimates of overlap. However, there are at least three reasons why this scenario is unlikely. First, we directly verified whether a given observed component contributed to performance in the task by regressing the component’s amplitude against the trial’s picture naming latency (see Fig. 6). These results revealed that all regions including those with activity across the entire epoch contributed to performance in the picture naming task. Second, note that regions with activity across the entire epoch were found in a wide variety of brain areas that included various parietal (IC5, IC10, IC24), occipital (IC9, IC13) and temporal regions (IC8, IC25). It seems unlikely that all these regions implement general cognitive processes that are not directly related to the picture naming task. Thus, even though the EEG method cannot conclusively establish causal relationships between activated regions and the involvement in the task^52^, it seems highly unlikely that the source regions that showed activity across an entire epoch are unrelated to the picture naming task. Moreover, as demonstrated above in the complementary analyses, even if we only consider transiently activated sources that are in each of the four major lobes, they are still activated concurrently for nearly half of the time that they are activated (45%). Thus, it seems unlikely that we have overestimated the temporal overlap of temporal profiles during the picture naming task.

The anatomical sources and their temporal dynamics observed in the current study correspond closely to those reported in previous studies. Previous hemodynamic studies of picture naming have typically reported activity in medial and lateral regions of the occipital cortex, in medial inferior and middle gyri of the temporal cortex, in posterior medial and inferior lateral portions of the parietal cortex, and in medial anterior (cingulate) and in medial and lateral (motor) regions of the frontal cortex^8,10–12,57,58^. The current study found activations in occipital (cuneus, lateral, fusiform), temporal (inferior, middle), frontal (medial cingulate, lateral), and parietal areas (inferior, superior) and thus align with those previously reported. Moreover, our study is in line with a recent EEG study examining the spatio-temporal dynamics of large-scale brain networks underlying the picture naming task^59^. Interestingly, this study reported various overlapping transient and stable large-scale brain networks underlying the task, and that strikingly, the occipital cortex remained activated across a series of such networks that spanned the initial 400 ms of picture naming. This observation of concurrent occipital cortex activity with other regions resonates well with our observation that IC9 (cuneus, central occipital) overlapped strongly with the other identified components. Thus, despite the limitations on localization accuracy due to fundamental properties of the EEG technique as well as due to the limited coverage available in the current study e.g.^60^, the source estimates and temporal dynamics obtained in our study are in line with those obtained in previous studies.

The data reported here suggest that brain regions involved in speech production display largely parallel activity. The finding of parallel activated brain regions in the domain of speech production fits well with data from other research domains. For example, early neurophysiological studies of intra-cranial recordings in monkey have revealed the near-instantaneous and parallel activity of many areas of the brain during manual motor decision tasks [e.g.,^61–63^ and see^64^ for an early review]. For example^62^, recorded local field potentials from 15 implanted electrodes in various cortical areas during a go/no-go task. They found evidence for a fast feedforward sweep of activity in occipital and frontal areas of the brain within 100 ms of stimulus presentation. Crucially, the go/no-go decision was found at around 150 ms and was evident in many different areas of the brain including occipital, parietal, temporal and pre-motor areas. These authors suggested that the decision to ’go’ was subserved by parallel activity in distinct brain regions. Similarly, fMRI studies have focused on task-based or resting-state networks that are assumed to reflect parallel activity across many different regions of the brain e.g.^65^. Finally, a large number of electrophysiological studies are currently exploring ideas about how ensembles of neurons in different areas of the brain communicate with each other and take the large-scale parallel nature of brain activity as a given e.g.^59,66^. Thus, our finding that speech production relies on the activation of parallel networks of brain regions is well-supported by other research findings across different research domains.

Two aspects of the results reported here warrant further discussion. First, it is worth pointing out the diversity of temporal profiles that underlie the production of speech. For example, for all ICs there is strong synchronized activity across trials, but for some this synchronized activity is of a rather short duration (IC5, 8, 9), for others this synchronized activity has a longer duration (IC6, 10, 11). Similarly, for some regions activity has a sharp rising edge while for others there is a more gradual slope (cf., IC5 and IC6). Such slow rising slopes in IC6 (caudal ACC) are reminiscent of the bereitshaft-potential typically observed over premotor cortices^67^. Whether these aspects of activation dynamics are meaningful is a question for future research. Second, complementary to the apparent diversity across temporal profiles, there are also regions that have quite similar temporal profiles (cf., IC5 and 8). Computing higher-order structure using functional connectivity analyses may provide further insight into how parallel computations underlie the production of speech (again see^59^ for similar observations).

One limitation of the current approach is that it relies on a group-level analysis rather than a more conventional participant-level analysis. Specifically, as described above, our analysis approach relied on the concatenation of all individual participant’s data matrices into one large data matrix. Subsequent analyses (ICA, ECD) were performed on this group level specification of the data that ignores differences between individual participants. The reason why we opted for this group-level approach is that a comparable approach at the individual participant level did not work well. Specifically, the data from individual participants was relatively noisy which meant we had problems finding clear source localizations with small errors for individual participants. As demonstrated here, combining the data across participants led to reliable source localizations with small residual error variances. The main drawback of the group level approach adopted here concerns the level at which statistical inferences can be specified. Whereas traditional by-participant analyses of EEG data permit the generalization of results to different participants that perform the same experiment (for discussion on this point see, for example^53^), the current results generalize to the current set of participants that performed this experiment. Future studies that either use this analysis approach at the individual participant level, or that enable back projection of group level ICA data to the individual participants [as is common in fMRI^68^;], could resolve the issues related to statistical inference present in the results obtained here.

A second limitation pertains to the range of timepoints for which our interpretation holds. Given that we have only analyzed the first 500 ms, our conclusions only hold for this time period and given that picture naming takes around 700 ms, it remains theoretically possible that brain activity proceeds in a sequential manner for the final 200 ms or so. However, although we currently cannot rule out this possibility, we deem it highly unlikely. A final limitation concerns the particular algorithm that was used for onset detection. We used a method in which we assumed that a source becomes active when it reaches the FWHM point (i.e., the time point at which its activation value passes 50% of the maximum value point). However, this choice is arbitrary and other criteria should be considered.

Before concluding one final issue needs to be addressed. As pointed out in the Introduction, there are a number of studies in the field of language production and EEG that have examined whether cognitive/linguistic components involved in speech production are activated in a serial or parallel fashion e.g.^4,20–25^. However, our current data have no bearing on the conclusions reached by these studies. Indeed as argued by^19^, even though brain activity in areas subserving speech production may be parallel, the cognitive and linguistic components that are computed by this parallel activity may nevertheless proceed in a serial manner (see discussion between^19,25^). For example, neurophysiological studies may show early activity in motor cortex during the production of speech, suggesting parallel processes. However, as pointed out by^19^, such early activity in motor cortex may reflect sequential semantic processing that relies on embodied representations. In other words, the current data on the dynamics of brain activity cannot be used to distinguish between theoretical proposals on the timecourse of cognitive and linguistic components during speech production. In our opinion, the question is whether theoretical proposals about the timecourse of cognitive/linguistic processes can generate falsifiable predictions that may be settled in the long run.

To conclude, speech production is a complex skill that relies on neural activity in many different areas of the brain. Previous studies using the MEG technique have claimed that speech production in the picture naming task progresses sequentially from occipital input areas to higher-level temporo-parietal and finally to frontal motor output areas e.g.^14,16,17^. Here we attempted to verify this claim using the EEG technique and a different analysis approach. Using group temporal ICA and ECD source modeling we identified 15 different regions of the brain that were verified to affect performance in the task, and which were previously identified in fMRI picture naming studies e.g.^8,9,11,12^. At odds with the results from the previous MEG studies, our results revealed that there was a large degree of temporal overlap in the activity in the fifteen regions during the course of producing speech. These results fit well with results from other research domains e.g.^62,64,66^, and suggest that speech production relies on the parallel interactions between many different brain regions. Understanding how these interactions lead to fast, fluent and non-pathological speech is a crucial question for future research.

## Supplementary information

Supplementary Materials.

## References

[1] Bressler SL (1995). Large-scale cortical networks and cognition. Brain Research Reviews.

[2] Chartier J, Anumanchipalli GK, Johnson K, Chang EF (2018). Encoding of articulatory kinematic trajectories in human speech sensorimotor cortex. Neuron.

[3] Fried I, Ojemann GA, Fetz EE (1981). Language-related potentials specific to human language cortex. Science.

[4] Indefrey P, Levelt WJ (2004). The spatial and temporal signatures of word production components. Cognition.

[5] Jürgens U (2002). Neural pathways underlying vocal control. Neuroscience & Biobehavioral Reviews.

[6] Piai V (2016). Direct brain recordings reveal hippocampal rhythm underpinnings of language processing. Proceedings of the National Academy of Sciences.

[7] Simonyan K, Horwitz B (2011). Laryngeal motor cortex and control of speech in humans. The Neuroscientist.

[8] Etard O (2000). Picture naming without brocaas and wernickeas area. Neuroreport.

[9] Geranmayeh F (2012). The contribution of the inferior parietal cortex to spoken language production. Brain and language.

[10] Janssen, N. & Mendieta, C. C. R. The dynamics of speech motor control revealed with time-resolved fmri. *Cerebral Cortex*, (2019). 10.1093/cercor/bhz084 31070731

[11] Murtha S, Chertkow H, Beauregard M, Evans A (1999). The neural substrate of picture naming. Journal of cognitive neuroscience.

[12] Price CJ (2012). A review and synthesis of the first 20 years of pet and fmri studies of heard speech, spoken language and reading. Neuroimage.

[13] Hulten A, Vihla M, Laine M, Salmelin R (2009). Accessing newly learned names and meanings in the native language. Human brain mapping.

[14] Liljeström M, Hulten A, Parkkonen L, Salmelin R (2009). Comparing meg and fmri views to naming actions and objects. Human brain mapping.

[15] Maess B, Friederici AD, Damian M, Meyer AS, Levelt WJ (2002). Semantic category interference in overt picture naming: Sharpening current density localization by pca. Journal of cognitive neuroscience.

[16] Salmelin R, Hari R, Lounasmaa O, Sams M (1994). Dynamics of brain activation during picture naming. Nature.

[17] Sörös P, Cornelissen K, Laine M, Salmelin R (2003). Naming actions and objects: cortical dynamics in healthy adults and in an anomic patient with a dissociation in action/object naming. Neuroimage.

[18] Vihla M, Laine M, Salmelin R (2006). Cortical dynamics of visual/semantic vs. phonological analysis in picture confrontation. Neuroimage.

[19] Indefrey P (2016). On putative shortcomings and dangerous future avenues: response to strijkers & costa. Language, Cognition and Neuroscience.

[20] Fargier R, Laganaro M (2016). Spatio-temporal dynamics of referential and inferential naming: Different brain and cognitive operations to lexical selection. Brain Topography.

[21] Kober H (2001). New approach to localize speech relevant brain areas and hemispheric dominance using spatially filtered magnetoencephalography. Human brain mapping.

[22] Miozzo M, Pulvermüller F, Hauk O (2014). Early parallel activation of semantics and phonology in picture naming: Evidence from a multiple linear regression meg study. Cerebral Cortex.

[23] Munding D, Dubarry A-S, Alario F-X (2016). On the cortical dynamics of word production: A review of the meg evidence. Language, Cognition and Neuroscience.

[24] Rahman RA, Sommer W (2003). Does phonological encoding in speech production always follow the retrieval of semantic knowledge?: Electrophysiological evidence for parallel processing. Cognitive Brain Research.

[25] Strijkers K, Costa A (2016). The cortical dynamics of speaking: Present shortcomings and future avenues. Language, Cognition and Neuroscience.

[26] Hämäläinen M, Hari R, Ilmoniemi RJ, Knuutila J, Lounasmaa OV (1993). Magnetoencephalography-theory, instrumentation, and applications to noninvasive studies of the working human brain. Reviews of modern Physics.

[27] Delorme A, Palmer J, Onton J, Oostenveld R, Makeig S (2012). Independent eeg sources are dipolar. PloS one.

[28] DiRusso F, Martínez A, Sereno MI, Pitzalis S, Hillyard SA (2002). Cortical sources of the early components of the visual evoked potential. Human brain mapping.

[29] Riès S, Janssen N, Burle B, Alario F-X (2013). Response-locked brain dynamics of word production. PLoS One.

[30] Glaser WR (1992). Picture naming. Cognition.

[31] Handy, T. C. *Event-related potentials: A methods handbook*. MIT press, (2005).

[32] Costa A, Strijkers K, Martin C, Thierry G (2009). The time course of word retrieval revealed by event-related brain potentials during overt speech. Proceedings of the National Academy of Sciences.

[33] Szekely A (2004). A new on-line resource for psycholinguistic studies. Journal of memory and language.

[34] Delorme A, Makeig S (2004). Eeglab: an open source toolbox for analysis of single-trial eeg dynamics including independent component analysis. Journal of neuroscience methods.

[35] Winkler, I., Debener, S., Müller, K.-R. & Tangermann, M. On the influence of high-pass filtering on ica-based artifact reduction in eeg-erp. In *Engineering in Medicine and Biology Society (EMBC), 2015 37th Annual International Conference of the IEEE*, pages 4101–4105. IEEE, (2015). 10.1109/EMBC.2015.7319296 26737196

[36] Acunzo DJ, MacKenzie G, van Rossum MC (2012). Systematic biases in early erp and erf components as a result of high-pass filtering. Journal of neuroscience methods.

[37] Maess B, Schröger E, Widmann A (2016). High-pass filters and baseline correction in m/eeg analysis. commentary on:how inappropriate high-pass filters can produce artefacts and incorrect conclusions in erp studies of language and cognition. Journal of neuroscience methods.

[38] Tanner D, Morgan-Short K, Luck SJ (2015). How inappropriate high-pass filters can produce artifactual effects and incorrect conclusions in erp studies of language and cognition. Psychophysiology.

[39] Widmann A, Schröger E, Maess B (2015). Digital filter design for electrophysiological data-a practical approach. Journal of neuroscience methods.

[40] Porcaro C, Medaglia MT, Krott A (2015). Removing speech artifacts from electroencephalographic recordings during overt picture naming. NeuroImage.

[41] Comon P (1994). Independent component analysis, a new concept?. Signal processing.

[42] Petersen, K., Hansen, L. K., Kolenda, T., Rostrup, E. & Strother, S. On the independent components of functional neuroimages. In *Third international conference on independent component analysis and blind source separation*, pages 615–620 (2000).

[43] Makeig S (2002). Dynamic brain sources of visual evoked responses. Science.

[44] Hyvarinen A (1999). Fast and robust fixed-point algorithms for independent component analysis. IEEE transactions on Neural Networks.

[45] Kovacevic N, McIntosh AR (2007). Groupwise independent component decomposition of eeg data and partial least square analysis. Neuroimage.

[46] Makeig S (2004). Electroencephalographic brain dynamics following manually responded visual targets. PLoS biology.

[47] Artoni F, Delorme A, Makeig S (2018). Applying dimension reduction to EEG data by principal component analysis reduces the quality of its subsequent independent component decomposition. NeuroImage.

[48] Makeig S, Debener S, Onton J, Delorme A (2004). Mining event-related brain dynamics. Trends in cognitive sciences.

[49] Desikan RS (2006). An automated labeling system for subdividing the human cerebral cortex on mri scans into gyral based regions of interest. Neuroimage.

[50] Delorme A, Sejnowski T, Makeig S (2007). Enhanced detection of artifacts in eeg data using higher-order statistics and independent component analysis. Neuroimage.

[51] Joyce CA, Gorodnitsky IF, Kutas M (2004). Automatic removal of eye movement and blink artifacts from eeg data using blind component separation. Psychophysiology.

[52] Weber MJ, Thompson-Schill SL (2010). Functional neuroimaging can support causal claims about brain function. Journal of cognitive neuroscience.

[53] Janssen N, Hernández-Cabrera JA, van der Meij M, Barber HA (2014). Tracking the time course of competition during word production: Evidence for a post-retrieval mechanism of conflict resolution. Cerebral Cortex.

[54] Guthrie D, Buchwald JS (1991). Significance testing of difference potentials. Psychophysiology.

[55] Protopapas A (2007). Check vocal: A program to facilitate checking the accuracy and response time of vocal responses from dmdx. Behavior Research Methods.

[56] Delorme A, Miyakoshi M, Jung T-P, Makeig S (2015). Grand average erp-image plotting and statistics: A method for comparing variability in event-related single-trial eeg activities across subjects and conditions. Journal of neuroscience methods.

[57] Geranmayeh F, Wise RJS, Mehta A, Leech R (2014). Overlapping networks engaged during spoken language production and its cognitive control. Journal of Neuroscience.

[58] Riecker A (2005). fmri reveals two distinct cerebral networks subserving speech motor control. Neurology.

[59] Hassan M (2015). Dynamic reorganization of functional brain networks during picture naming. Cortex.

[60] Song J (2015). Eeg source localization: sensor density and head surface coverage. Journal of neuroscience methods.

[61] Cisek P, Kalaska JF (2010). Neural mechanisms for interacting with a world full of action choices. Annual review of neuroscience.

[62] Ledberg A, Bressler SL, Ding M, Coppola R, Nakamura R (2006). Large-scale visuomotor integration in the cerebral cortex. Cerebral cortex.

[63] Liang H, Bressler SL, Ding M, Truccolo WA, Nakamura R (2002). Synchronized activity in prefrontal cortex during anticipation of visuomotor processing. Neuroreport.

[64] Fetz EE (1992). Are movement parameters recognizably coded in the activity of single neurons?. Behavioral and Brain Sciences.

[65] Smith SM (2013). Functional connectomics from resting-state fmri. Trends in cognitive sciences.

[66] Fries P (2005). A mechanism for cognitive dynamics: neuronal communication through neuronal coherence. Trends in Cognitive Sciences.

[67] Kornhuber HH, Deecke L (2016). Brain potential changes in voluntary and passive movements in humans: readiness potential and reafferent potentials. Pflügers Archiv-European Journal of Physiology.

[68] Nickerson, L. D., Smith, S. M., Öngür, D. & Beckmann, C. F. Using dual regression to investigate network shape and amplitude in functional connectivity analyses. *Frontiers in Neuroscience* (11, mar 2017). 10.3389/fnins.2017.00115 PMC5346569 28348512

