# Supplementary material for: Exploring the temporal dynamics of speech production with EEG and group ICA
Source: Sci Rep. 2020 Feb 28;10:3667. doi: 10.1038/s41598-020-60301-1 (PMC7048769; doi:10.1038/s41598-020-60301-1)
Supplement: Supplementary file 1 — Supplementary Materials. [file 41598_2020_60301_MOESM1_ESM.pdf]

Supplementary Materials for: Exploring the temporal dynamics of speech production with EEG and group ICA

Niels Janssen[1,2,3], Maartje van der Meij[1], Pedro Javier López-Pérez[4] and Horacio A. Barber-Friend[1,2,3,5]

[1]Departamento de Psicología, Universidad de la Laguna, Spain

[2]Instituto de Tecnologías Biomedicas, Universidad de la Laguna, Spain

[3]Instituto de Neurociencias, Universidad de la Laguna, Spain

[4]Departamento de Psicología Evolutiva y de la Educación, Universidad de Málaga, Spain

[5]Basque Center on Cognition, Brain and Language (BCBL), Spain

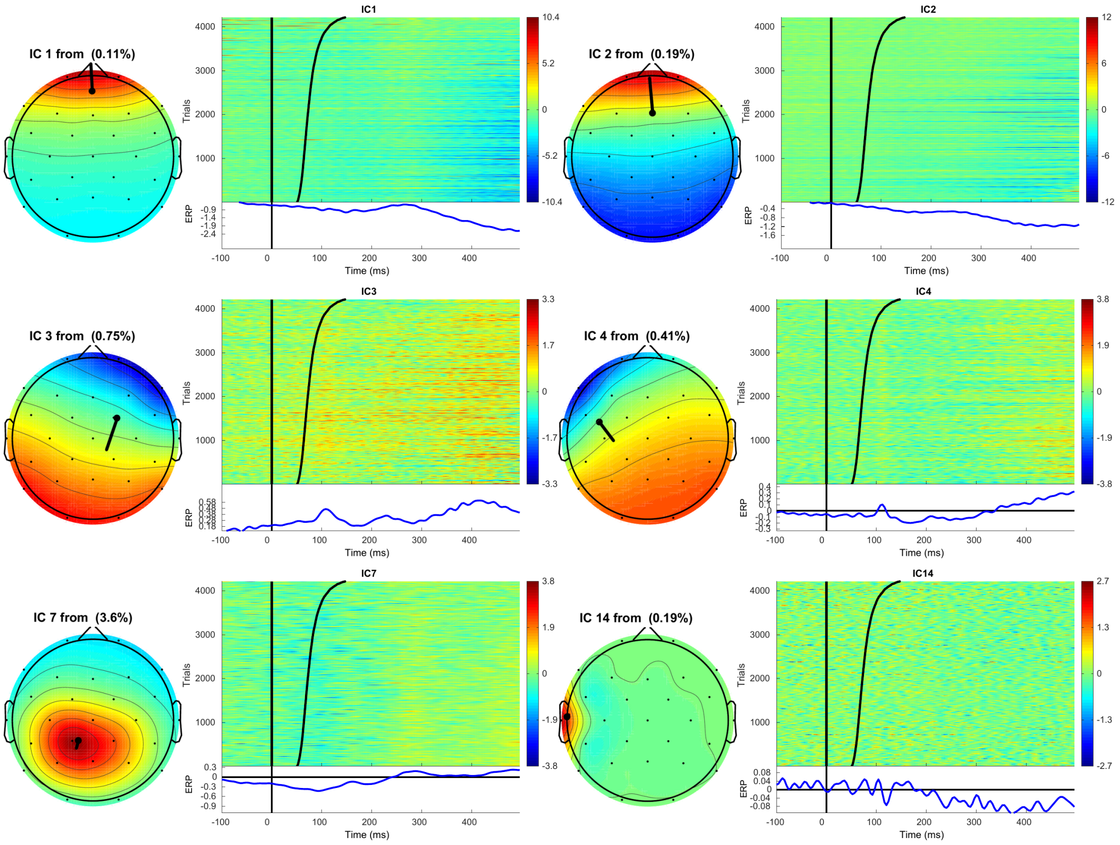

Figure S1: List of excluded components in range IC1-14. Note these components represent various noise components like eye blinks (e.g., ICs 1, 2), horizontal eye movements (e.g., ICs 3 and 4), and muscle artefacts (IC14).

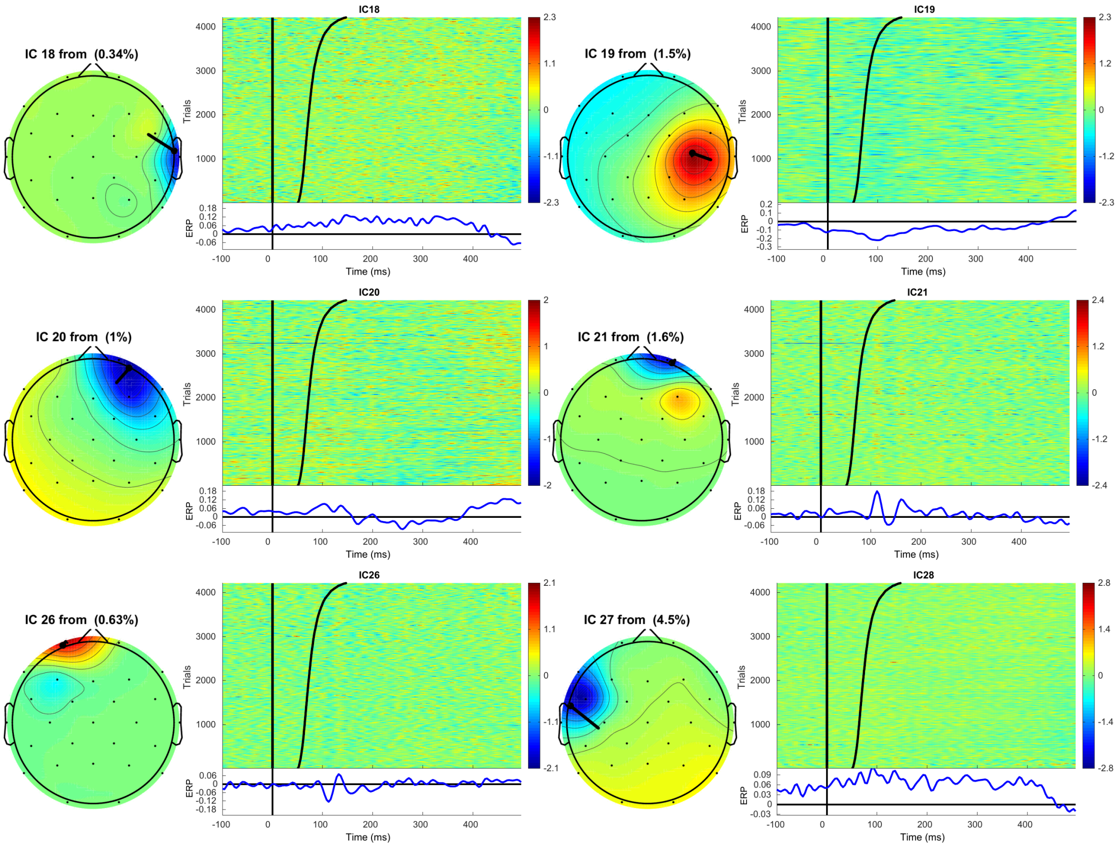

Figure S2: List of excluded components in range IC18-27. Note some components have multiple dipoles (e.g., ICs 21, 26)

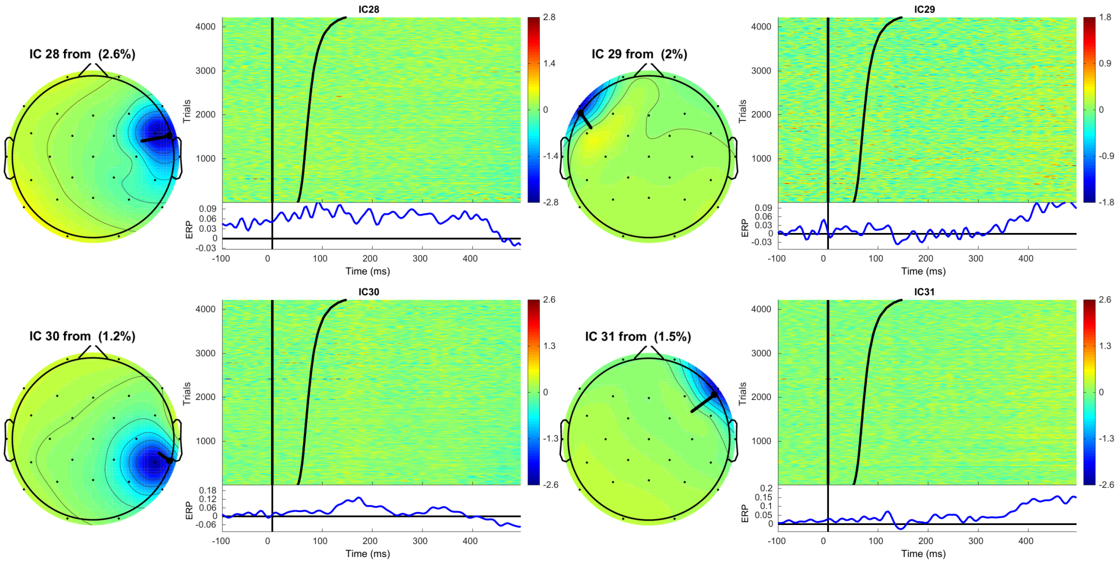

Figure S3: List of excluded components in range IC28-31. Note absence of consistent synchronized activity across trials.

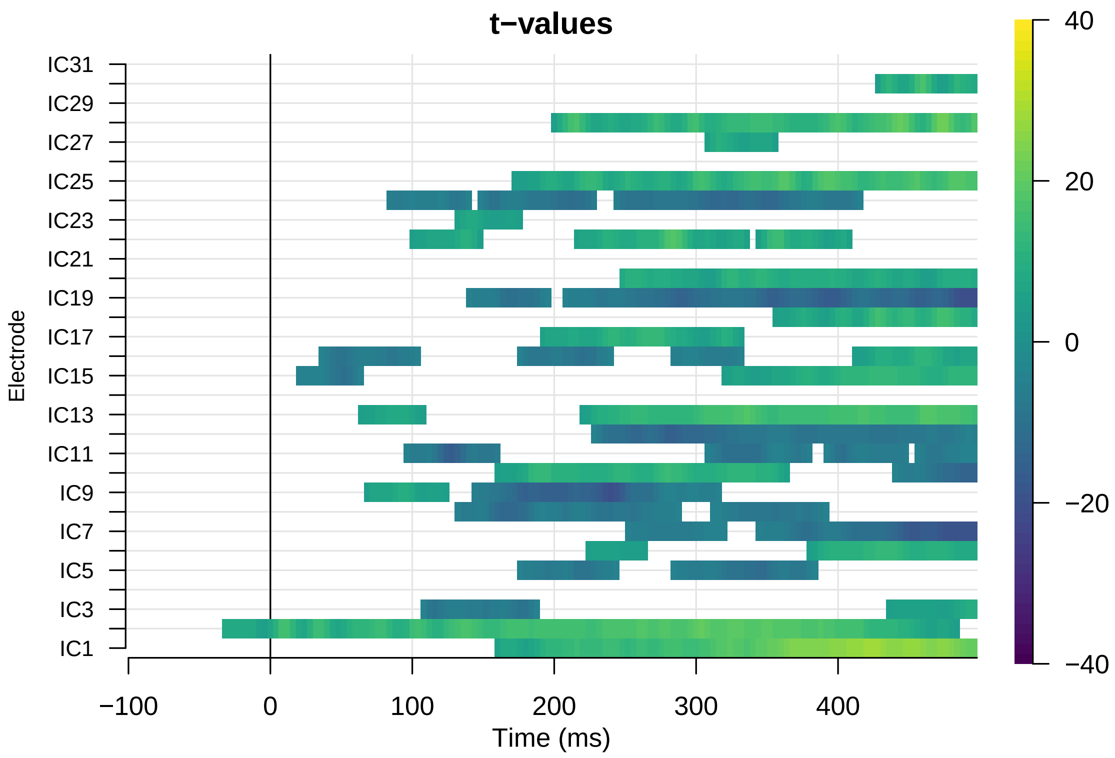

Figure S4: Overview of correlation between amplitudes and naming latencies for all obtained components.

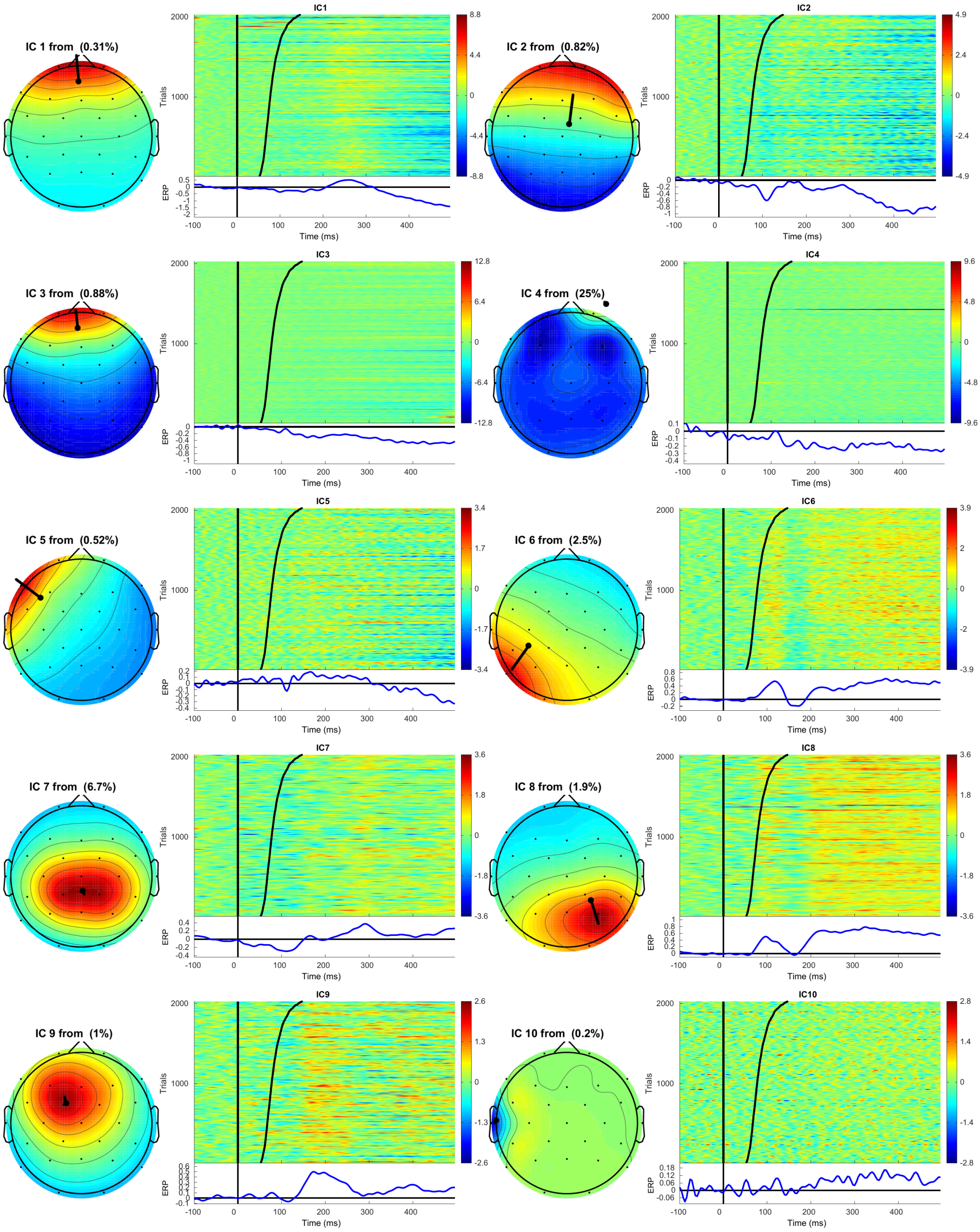

Figure S5: List of ICs 1-10 using only data from before familiarization.

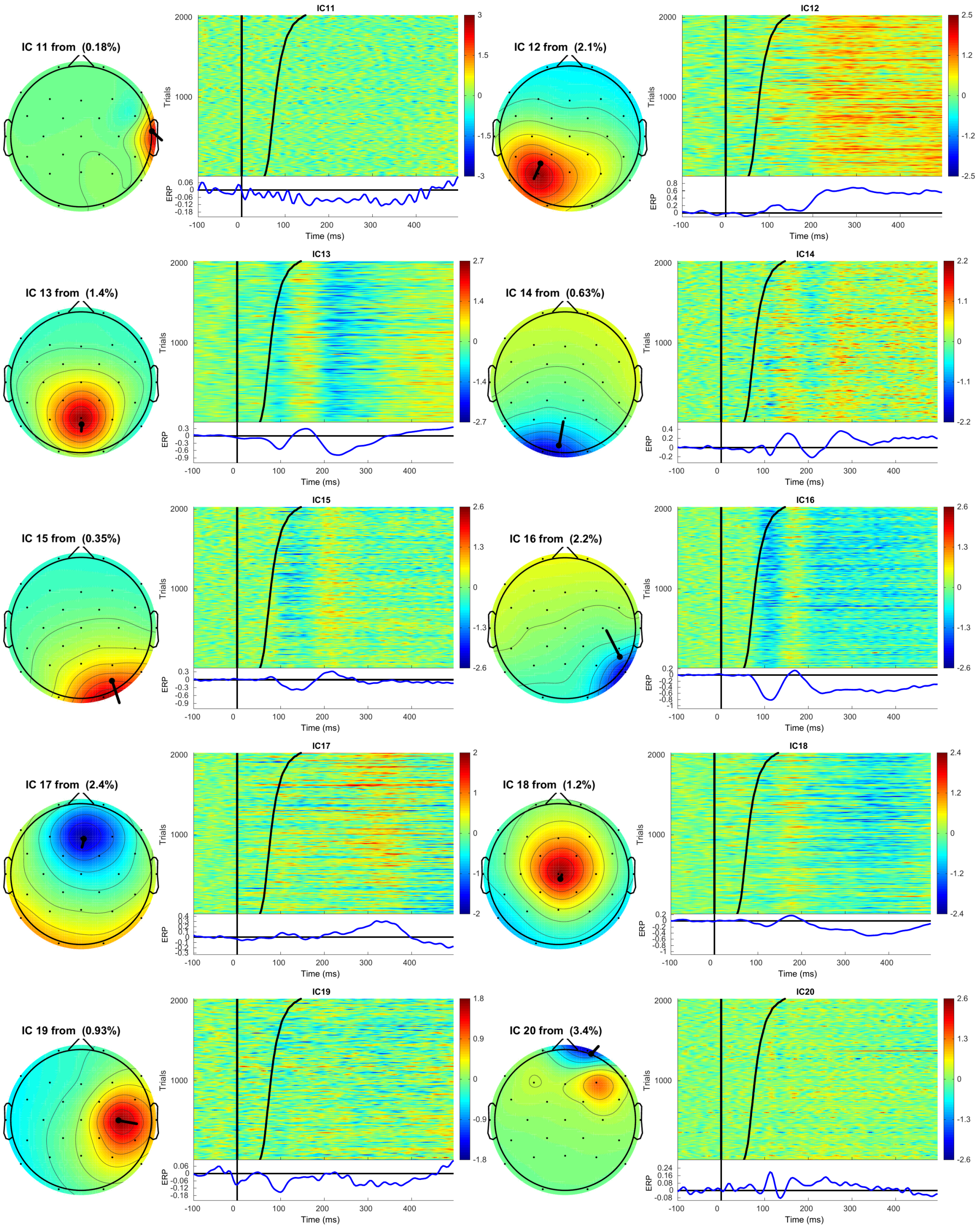

Figure S6: List of ICs 11-20 using only data from before familiarization.

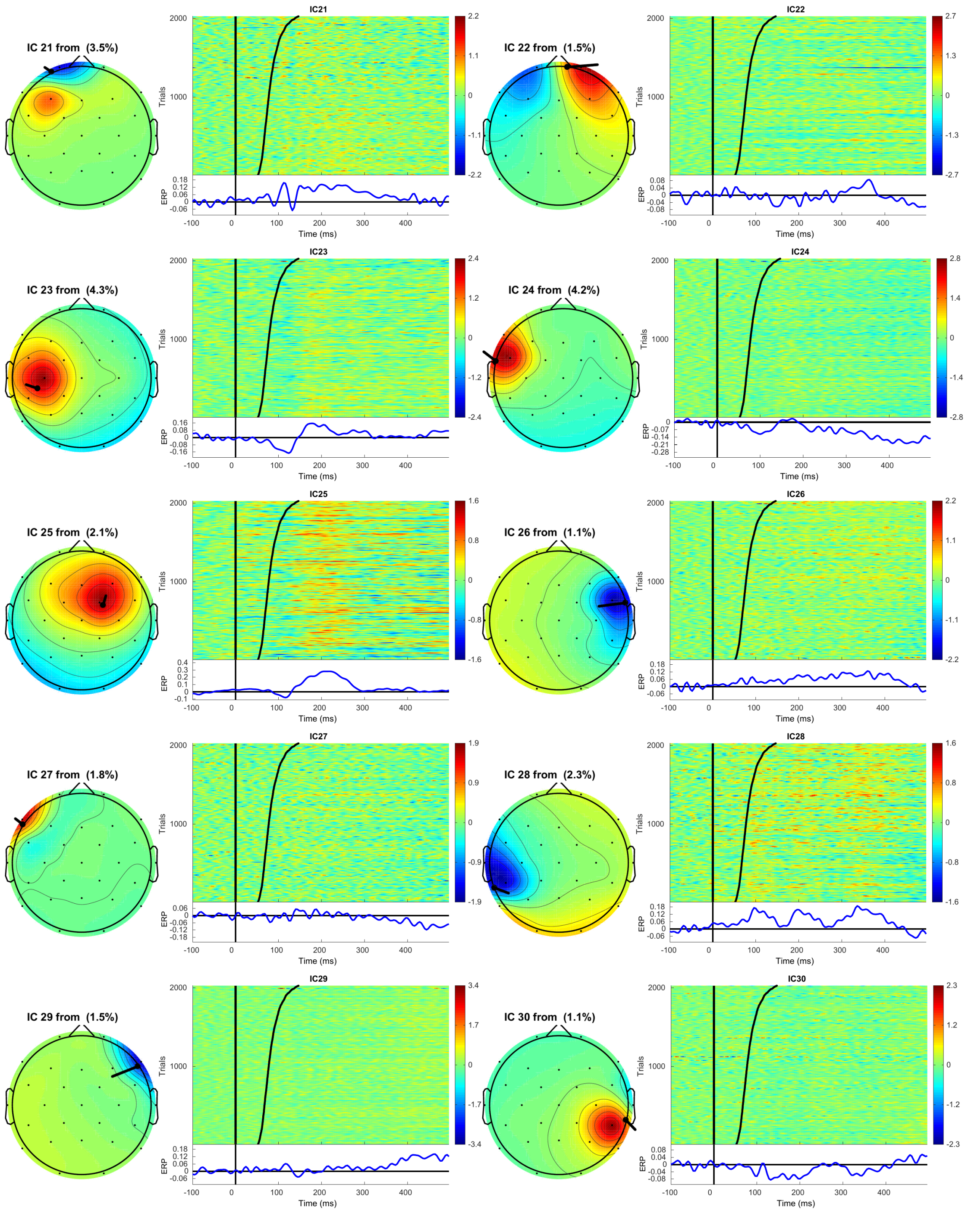

Figure S7: List of ICs 21-30 using only data from before familiarization.

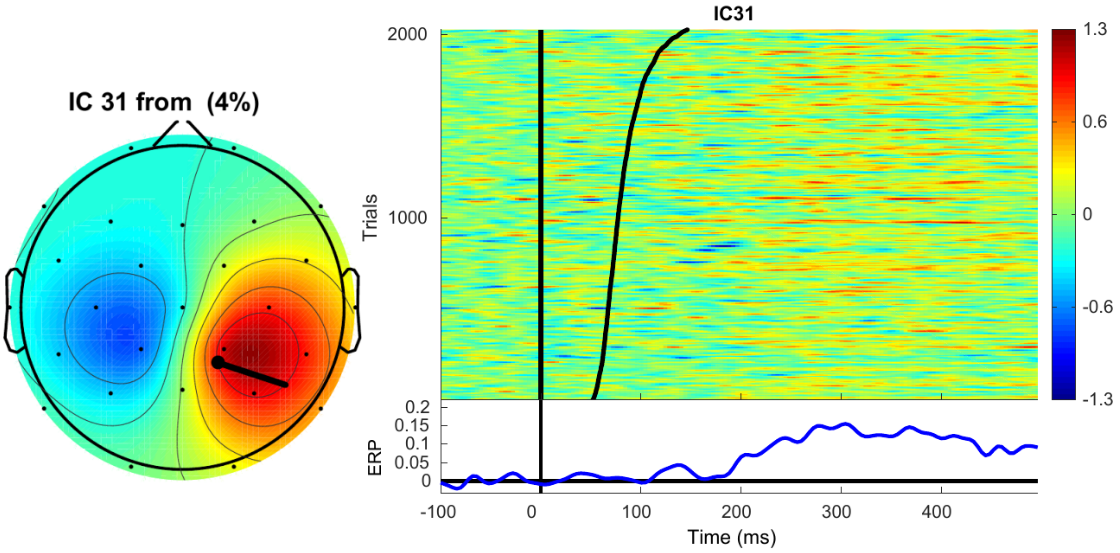

Figure S8: List of IC31 using only data from before familiarization.

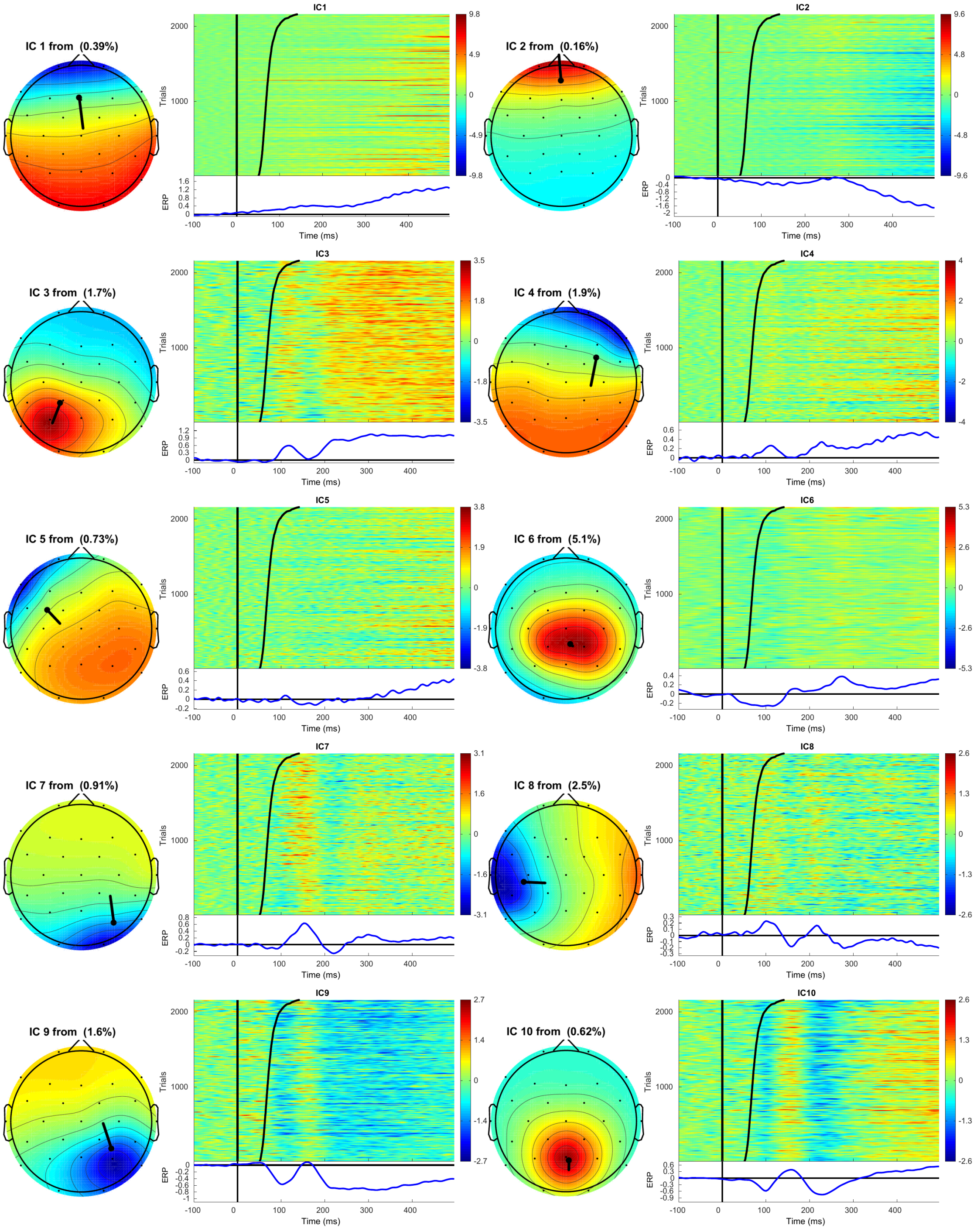

Figure S9: List of ICs 1-10 using only data from after familiarization.

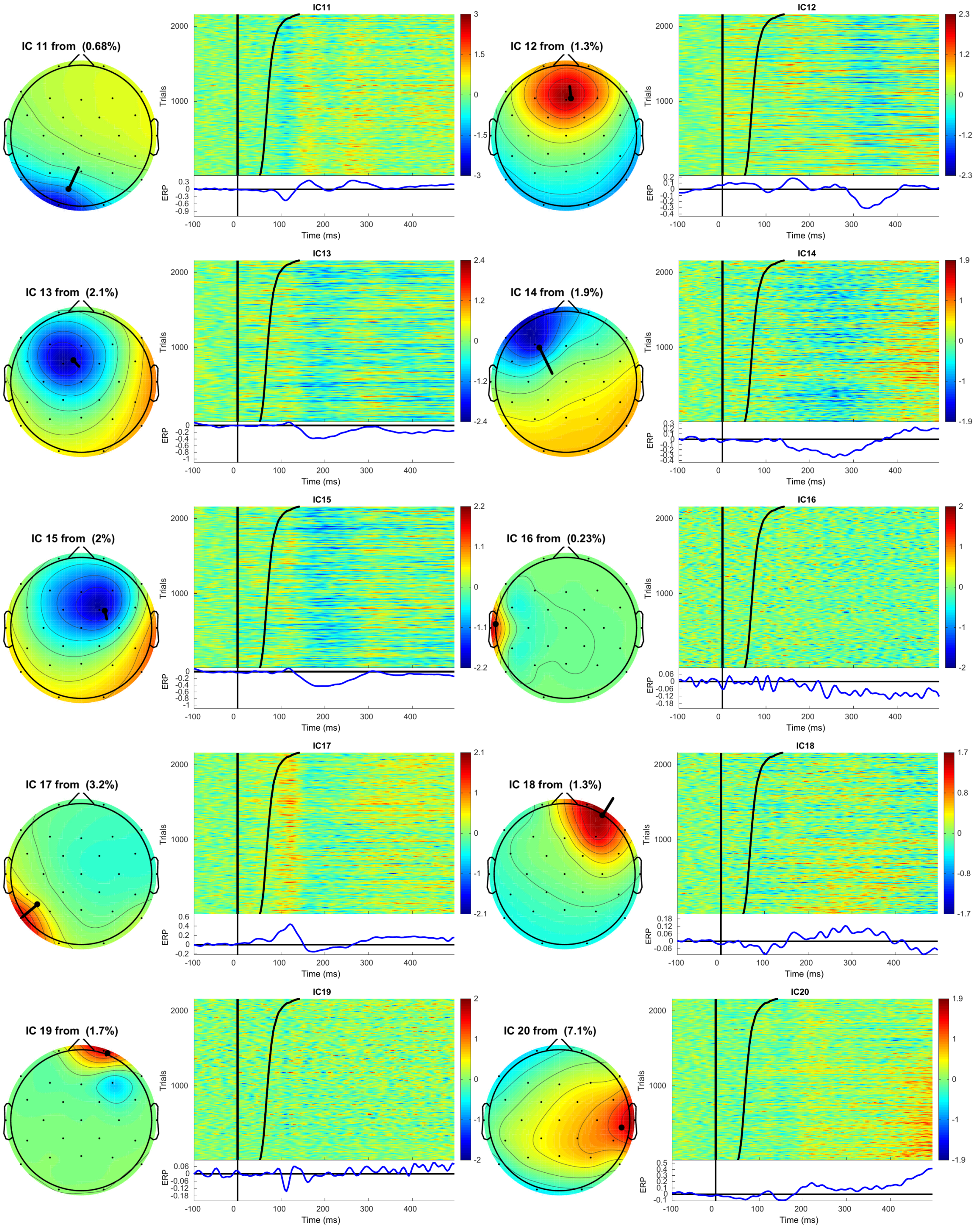

Figure S10: List of ICs 11-20 using only data from after familiarization.

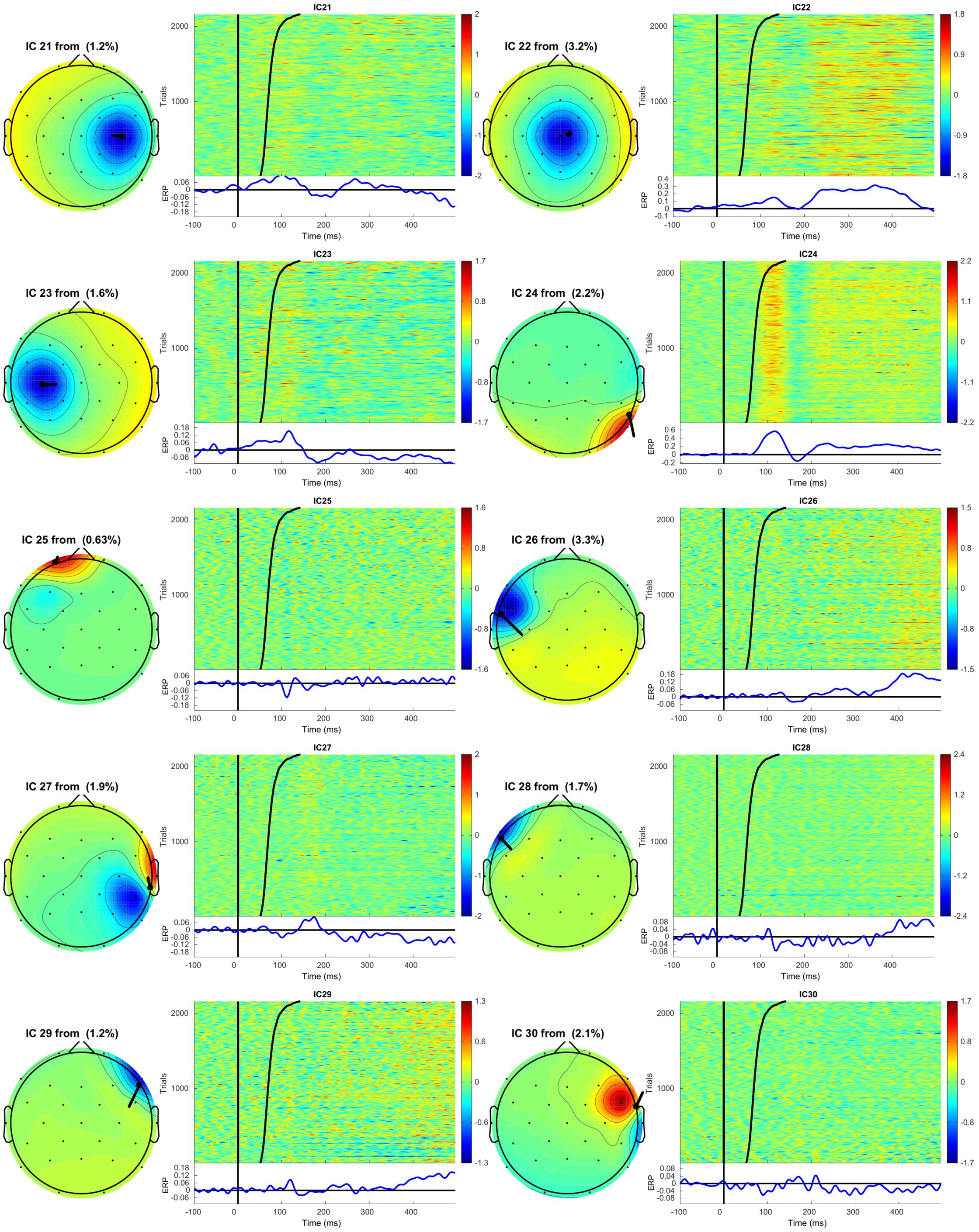

Figure S11: List of ICs 21-30 using only data from after familiarization.

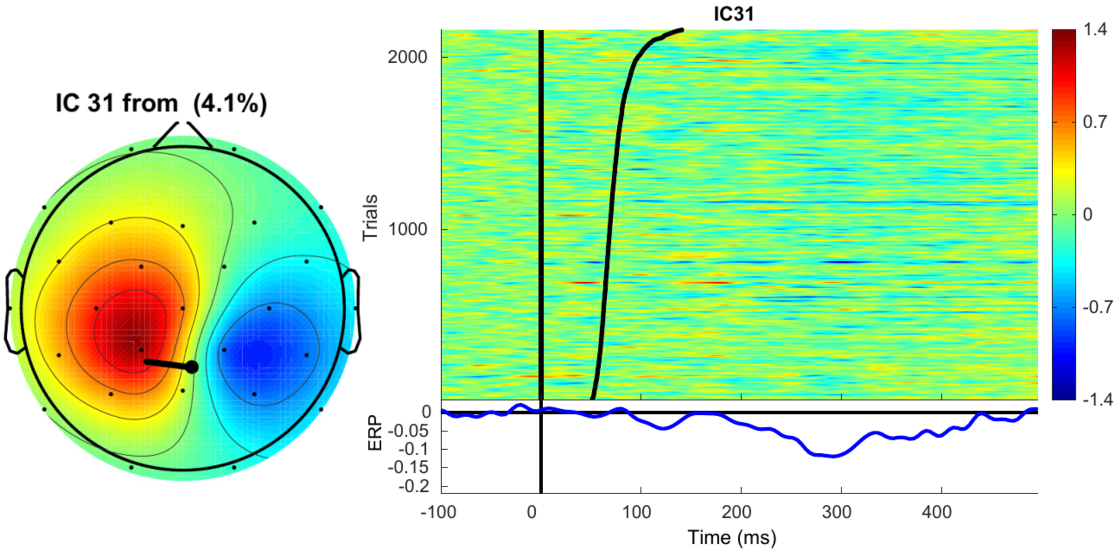

Figure S12: List of IC31 using only data from after familiarization.

Table S1: Ratios (top panel) and milliseconds (bottom panel) of group-level activity overlap between the time courses of for Independent Component from before familiarization.

	6	9	13	14	15	16	17	18	25
6	1.00	1.00	0.96	1.00	1.00	0.98	1.00	1.00	1.00
9	0.62	1.00	0.69	0.70	0.62	0.72	0.57	0.74	1.00
13	0.50	0.57	1.00	0.43	1.00	0.60	0.09	0.23	1.00
14	0.89	1.00	0.75	1.00	0.72	0.85	1.00	1.00	1.00
15	0.32	0.32	0.62	0.26	1.00	0.37	0.00	0.00	0.69
16	0.84	1.00	1.00	0.82	1.00	1.00	0.71	0.89	1.00
17	0.55	0.51	0.10	0.62	0.00	0.46	1.00	0.87	0.00
18	0.52	0.62	0.23	0.59	0.00	0.54	0.82	1.00	0.15
25	0.26	0.41	0.50	0.29	0.56	0.30	0.00	0.08	1.00
6	400.00								
9	248.00	248.00							
13	196.00	140.00	204.00						
14	356.00	248.00	152.00	356.00					
15	124.00	76.00	124.00	88.00	124.00				
16	336.00	248.00	204.00	292.00	124.00	344.00			
17	220.00	124.00	16.00	220.00	0.00	156.00	220.00		
18	208.00	152.00	44.00	208.00	0.00	184.00	180.00	208.00	
25	100.00	100.00	100.00	100.00	68.00	100.00	0.00	12.00	100.00

Table S2: Ratios (top panel) and milliseconds (bottom panel) of group-level activity overlap between the time courses of for Independent Components from after familiarization.

	7	8	9	10	11	12	13	14	15	17	22	24
7	1.00	1.00	1.00	0.94	0.85	1.00	1.00	1.00	1.00	1.00	0.94	1.00
8	0.98	1.00	1.00	0.92	0.84	1.00	1.00	1.00	1.00	1.00	0.92	1.00
9	0.60	0.61	1.00	0.56	0.51	0.68	0.69	0.70	0.62	0.57	0.56	0.62
10	1.00	1.00	1.00	1.00	0.91	1.00	1.00	1.00	1.00	1.00	1.00	1.00
11	1.00	1.00	1.00	1.00	1.00	1.00	1.00	1.00	1.00	1.00	1.00	1.00
12	0.70	0.72	0.79	0.66	0.60	1.00	0.44	0.82	0.22	1.00	0.66	0.73
13	0.50	0.50	0.57	0.46	0.42	0.31	1.00	0.43	1.00	0.09	0.46	0.50
14	0.86	0.87	1.00	0.80	0.73	1.00	0.75	1.00	0.72	1.00	0.80	0.88
15	0.30	0.31	0.32	0.29	0.26	0.09	0.62	0.26	1.00	0.00	0.29	0.31
17	0.53	0.54	0.51	0.50	0.46	0.76	0.10	0.62	0.00	1.00	0.50	0.55
22	1.00	1.00	1.00	1.00	0.91	1.00	1.00	1.00	1.00	1.00	1.00	1.00
24	0.97	0.99	1.00	0.91	0.83	1.00	0.98	1.00	1.00	1.00	0.91	1.00
7	416.00											
8	408.00	408.00										
9	248.00	248.00	248.00									
10	416.00	408.00	248.00	444.00								
11	416.00	408.00	248.00	444.00	488.00							
12	292.00	292.00	196.00	292.00	292.00	292.00						
13	204.00	204.00	140.00	204.00	204.00	88.00	204.00					
14	356.00	356.00	248.00	356.00	356.00	292.00	152.00	356.00				
15	124.00	124.00	76.00	124.00	124.00	24.00	124.00	88.00	124.00			
17	220.00	220.00	124.00	220.00	220.00	220.00	16.00	220.00	0.00	220.00		
22	416.00	408.00	248.00	444.00	444.00	292.00	204.00	356.00	124.00	220.00	444.00	
24	404.00	404.00	248.00	404.00	404.00	292.00	200.00	356.00	124.00	220.00	404.00	404.00
